# FIGARO-E3: a high-resolution extended multi-regional input-output database consistent with official statistics

**DOI:** 10.1038/s41597-025-04431-z

**Published:** 2025-04-04

**Authors:** Ignacio Cazcarro, Arkaitz Usubiaga-Liaño, Marίa Victoria Román, Pablo Piñero, Erik Dietzenbacher, José Manuel Rueda-Cantuche, Iñaki Arto

**Affiliations:** 1 Basque Centre for Climate Change, Parque Científicio de UPV/EHU, 48940 Leioa, Spain; 2 ARAID (Aragonese Agency for Research and Development), Agrifood Institute of Aragon (IA2), Department of Economic Analysis, Faculty of Economics and Business Studies, University of Zaragoza, 50005 Zaragoza, Spain; 3 Joint Research Centre (European Commission), 41092 Seville, Spain; 4 University of Groningen, Department of Global Economics & Management, Faculty of Economics and Business, Groningen, The Netherlands

**Keywords:** Environmental impact, Economics

## Abstract

Existing ‘official’ multi-regional input-output (MRIO) databases lack sufficient sectoral detail and extensions for calculating accurate environmental and social footprints. FIGARO-E3 is a highly disaggregated MRIO database for 2015 with labour and environmental extensions largely consistent with official statistics. The database has been created by disaggregating the official FIGARO database (46 countries, 64 industries/products) to achieve a resolution of 175 industries and 213 products based on the monetary structures of EXIOBASE. Labour accounts (including total employment and employment by gender and by skill) are based on OECD data and EXIOBASE structures. Energy accounts (primary energy supply, net, final and non-energy uses, energy industry own use and energy losses) are based on the IEA’s extended energy balances and FIGARO-E3 MRIO tables. GHG emission accounts (covering four types of GHGs: CO2, CH4, N2O and fluorinated gases, both for combustion and non-combustion processes) are based on IEA and EDGAR data. GHG emission accounts for European countries have been reconciled with data from Eurostat. The FIGARO-E3 database is largely consistent with official statistics.

## Background & Summary

The last decades have seen a rapid increase of traded goods and services, as well as international movement of capital and people due to the globalization process^1^. This is evident when looking at, for example, the value of trade of goods and services as a percentage of world’s GDP^2^, the rise of foreign direct investment, the stock of international claims as a percentage of world’s GDP, geo-economic fragmentation of production^3,4^, or processes of offshoring production^5–7^. The term globalization sometimes refers to the movement of people (labour) and knowledge (technology) across international borders, but there are broader cultural, political, and environmental implications of globalization. In the environmental dimension, globalization has been associated with an increase of carbon emissions and biodiversity threats^8,9^, but the increase of negative consequences is not limited to these areas and extends to other environmental and social areas such as air and water pollution, water scarcity and child labour^10^.

Assessing the social, economic and environmental implications of globalization requires addressing production, consumption and trade patterns. For example, from the environmental perspective, countries often set the main focus on technology (the production side), but increasing affluence, which leads to increasing consumption, is considered a key driving factor behind current trends in environmental degradation^11^. This holds in a similar way for other areas. Consumption (and increases therein) in one country may have serious affects in another country. The need for information on the consumption perspective has led to a considerable increase in the number of academic papers devoted to calculate different types of footprints, which make the link between final consumption in one country/region and social or environmental impacts worldwide^12^. Also, there is a growing interest in the scientific^13^ and policy^14,15^ communities about the social and economic impact of trade in national economies and, in particular, about how this impact is linked to their role in global value chains.

These analyses are typically conducted using multiregional input-output (MRIO) databases with environmental and social extensions. Currently, a wide variety of MRIO databases exist, each of which with different features and built using different methodological approaches and data sources. Most of these databases have been built by scientist in the framework of research projects (the most widely used ones are described in^16,17^), although a couple of exceptions exist that are built by statistical offices^18,19^. Multi-database comparisons have reported relevant differences between the main MRIO databases with respect to their monetary structure; their extensions; and their footprint results^20–28^. The results are also affected by different sectoral^29–32^ and geographical resolutions^33^. It is important to note though that the main databases agree on carbon footprint trends for most major regions^27^.

Despite the efforts behind the construction of and comparison between different MRIO databases, the policy uptake of the results is limited. The key problem seems a lack of MRIO databases that (1) have a sufficient amount of detail in terms of industries and products and in terms of (social and environmental) extensions, and (2) are consistent with national statistics, increasing the credibility of the results. The need for such databases has been recognised by several researchers with a call for international institutions to take the lead in their production^34^. While the OECD and Eurostat have produced their own MRIO databases^18,19^, they still lack sufficient sectoral detail and environmental and social extensions to more accurately represent environmental and social footprints. For instance, having a single agricultural sector is problematic when calculating carbon footprints because the emission profiles of the underlying products are very different (e.g. rice, vegetables, red meat from ruminants, etc.), which links back to the aggregation bias problem referred to previously. For this reason, the need to add additional sectoral detail to these databases has been encouraged^34^.

One approach to handle the key problem has, in some countries, been to combine detailed official data produced by national statistical offices with existing MRIO databases^35–38^. This approach works well when the focus is on single a country and some global aspects need to be added. Because our focus is not on a particular, single country, we follow a different approach. Namely, by taking an existing MRIO database that is consistent with national statistics and adding detail, whilst preserving consistency.

To this end, the European Commission’s Joint Research Centre funded a project to build a high-resolution MRIO database based on Eurostat’s FIGARO database^39^, which was to include energy, air emission and labour extensions. The main novelty of FIGARO-E3 (E3 meaning employment, energy and emissions) is that it combines detailed information and consistency with official statistics, thus substantially increasing its potential use in policy. This first version of the database covers the year 2015, and represents 46 countries (plus a rest of the world region), 176 industries and 213 products. It includes five energy extensions, four types of greenhouse gas (GHG) emissions, and two labour extensions. Energy extensions include data on primary energy supply, gross energy supply, gross energy use, net energy use, final energy use, energy industry own use, energy losses, non-energy use and emission relevant energy use. GHG emission extensions provide information on the emission of four GHG categories: carbon dioxide (CO_2_), methane (CH_4_), nitrous oxide (N_2_O) and fluorinated gases (F-gases). Labour accounts represent total employment (jobs/persons) by gender (male and female) and by three skill levels (low, medium and high). Monetary data is reported in Million €, energy accounts in Terajoules; CO_2_, CH_4_ and N_2_O in kilotons (kt) and F-gases are given in kt CO_2_e (using Global Warming Potential factors to 100 years from^40^).

The following sections describe FIGARO-E3 in more detail and provide instructions on how to use it and interpret the results.

## Methods

**Fig. 1:**
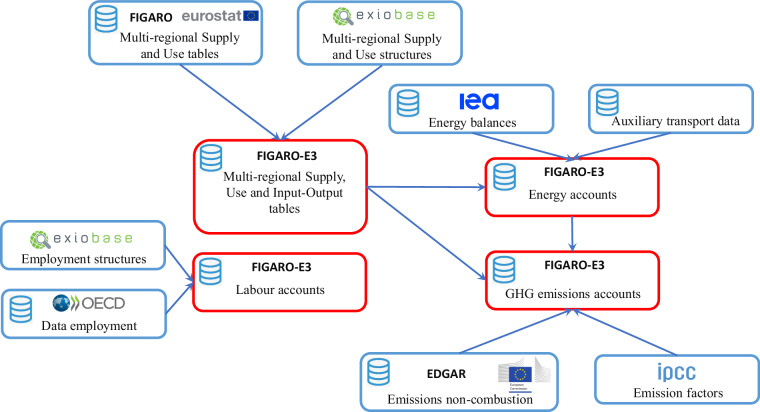
Summary of FIGARO-E3 and the inputs from other datasets.

Figure 1 summarises the construction of the database including the main inputs (in blue) and outputs (in red).

The structure of the database can be split in four main blocks. In the first block, we find the rectangular FIGARO-E3 Multi-Regional Supply and Use Tables (MRSUTs), from which the Multi-Regional Input-Output Tables (MRIOTs) are derived. These tables are built departing from Eurostat’s FIGARO database^39^. The original 64 industries and 64 products in FIGARO are disaggregated into 176 industries and 213 products using the structures of the EXIOBASE MRSUTs^41,42^.

The labour accounts represent the second block of the FIGARO-E3 database. It starts from the highly-aggregated (a maximum of 36 industries) OECD labour data by gender and skill^43^, which is disaggregated into 176 industries using EXIOBASE labour and output data^41,42^.

The third block are energy accounts, which are based on the IEA energy balances^44,45^. Combining them with a series of auxiliary transport datasets (as described below) and the FIGARO-E3 MRSUTs yields the energy accounts.

The last block contains the GHG emission accounts. For emissions from combustion, it combines energy data from the IEA energy balances^44,45^ with emission factors from the Intergovernmental Panel on Climate Change^46^. For emissions not related to combustion, we use the emission data from non-combustion processes from the EDGAR database^47^. All of emissions are disaggregated, if necessary, using the FIGARO-E3 MRSUTs. Data for the European countries is rescaled to official Eurostat data^48^, i.e. it is made consistent with European emission accounts at 64-industry level.

Next, we summarise the methods for the construction of each of these blocks. Detailed information on the methods is reported in the supplementary material (SM).

### Multi-regional supply, use and input-output tables

The original FIGARO MRSUTs were disaggregated to fit the classification of FIGARO-E3, based on the structures of EXIOBASE MRSUTs. Before doing so, we performed comparisons between FIGARO and EXIOBASE databases, in line with the comparisons we show with several databases in the Technical Validation. For the construction we aim to combine the best of both worlds. On the one hand, the industry and product classification of FIGARO-E3 adopts the high detail of EXIOBASE in agriculture, mining, manufacturing and energy sectors. In this sense, despite the inaccuracies that EXIOBASE might have, for footprint analyses it is considered preferable to disaggregate heterogenous sectors even if partial information is used^49,50^. On the other hand, it adopts the very detailed structure for the service sector from FIGARO. The full classification is shown in the SM. The process of disaggregating FIGARO is undertaken in three steps: an initial split, a bi-proportional balancing process leading to MRSUTs and the transformation of the MRSUTs into MRIOTs.

**Fig. 2:**
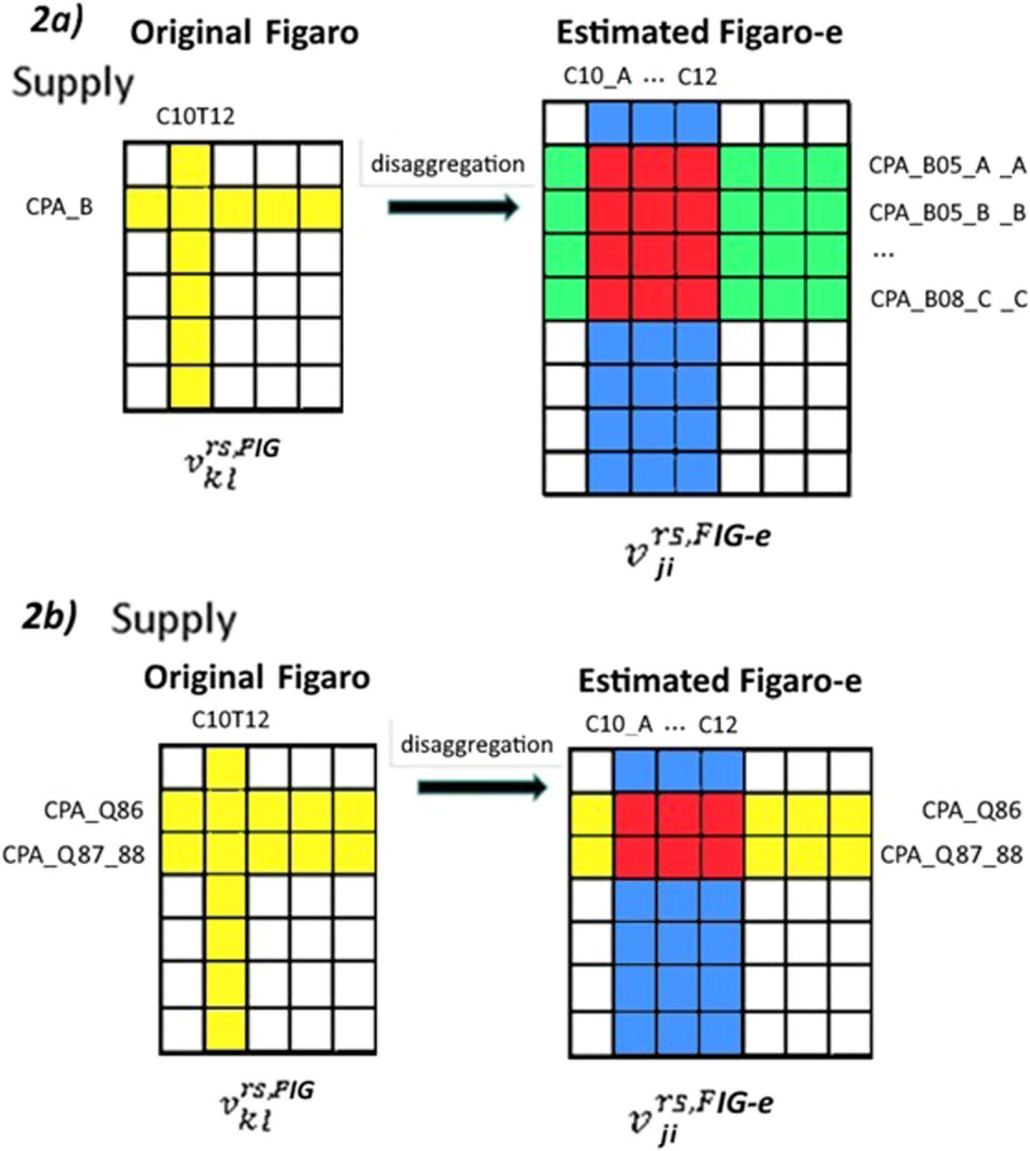
Graphical representation of the initial split of FIGARO Supply (in the example) and Use tables.

In the first step we provide an initial estimate with two basic cases. The first case is where one industry and/or product in FIGARO corresponds to several industries/products in EXIOBASE. In that case, FIGARO-E3 disaggregates the FIGARO data using the shares from EXIOBASE. Assume that industry *l* in FIGARO corresponds to industries $i={l}_{1},\ldots ,{l}_{p}$ in EXIOBASE and/or that product *k* in FIGARO corresponds to products $j={k}_{1},\ldots ,{k}_{q}$. In that case, we have

$$\begin{array}{cc}{\bar{v}}_{{ji}}^{{\rm{FIG}}-{\rm{e}}}={v}_{{kl}}^{{\rm{FIG}}}\frac{{v}_{{ji}}^{{\rm{EXIO}}}}{{\sum }_{i={l}_{1}}^{{l}_{p}}{\sum }_{j={k}_{1}}^{{k}_{q}}{v}_{{ji}}^{{\rm{EXIO}}}},\, & {\rm{for}}\,i={l}_{1},\ldots ,{l}_{p}\,{\rm{and}}\,j={k}_{1},\ldots ,{k}_{q}\end{array}$$

(the red part in Fig. 2a)

$$\begin{array}{cc}{\bar{v}}_{{ji}}^{{\rm{FIG}}-{\rm{e}}}={v}_{{kl}}^{{\rm{FIG}}}\frac{{v}_{{ij}}^{{\rm{EXIO}}}}{{\sum }_{j={k}_{1}}^{{k}_{q}}{v}_{{ji}}^{{\rm{EXIO}}}},\, & {\rm{for}}\,i\ne {l}_{1},\ldots ,{l}_{p}\,{\rm{and}}\,j={k}_{1},\ldots ,{k}_{q}\end{array}$$

(the green part in Fig. 2a)

$$\begin{array}{cc}{\bar{v}}_{{ji}}^{{\rm{FIG}}-{\rm{e}}}={v}_{{kl}}^{{\rm{FIG}}}\frac{{v}_{{ji}}^{{\rm{EXIO}}}}{{\sum }_{i={l}_{1}}^{{l}_{p}}{v}_{{ji}}^{{\rm{EXIO}}}},\, & {\rm{for}}\,i={l}_{1},\ldots ,{l}_{p}\,{\rm{and}}\,j\ne {k}_{1},\ldots ,{k}_{q}\end{array}$$

(the blue part in Fig. 2a).

Here, ${v}_{{ji}}^{{\rm{EXIO}}}$ gives the supply of product *j* by industry *i* in EXIOBASE, ${\bar{v}}_{{ji}}^{{\rm{FIG}}-{\rm{e}}}$ gives the estimate (indicated by an overbar) in FIGARO-E3, and ${v}_{{kl}}^{{\rm{FIG}}}$ gives the aggregated supply in FIGARO. The same procedure is used to estimate the use of product *j* in industry *i* (i.e., ${\bar{u}}_{{ji}}^{{\rm{FIG}}-{\rm{e}}}$). In the example of Fig. 2a, industry C10T12 in FIGARO is split into 12 industries in FIGARO-E3 (C10_A, …, C10_J, C11, C12) following the EXIOBASE classification. Also, product CPA_B in FIGARO is in FIGARO-E3 split into 23 products (CPA_B05_A, …, CPA_B05_H, CPA_B06_A, …, CPA_B06_D, CPA_B07_A, …, CPA_B07_H, CPA_B08_A, …, CPA_B08_C) following the EXIOBASE classification.

The second case is where several industries and/or products in FIGARO correspond to one industry/product in EXIOBASE. In that case, we adopt the detailed information from FIGARO and where necessary, we take the shares for the aggregated EXIOBASE industries/products in the process of disaggregation. Assume that the aggregate product *r* in EXIOBASE corresponds to products $j={r}_{1},\ldots ,{r}_{s}$ in FIGARO (see the yellow columns in Fig. 2b). The industry *l* in FIGARO, however, needs to be disaggregated into industries ${l}_{1},\ldots ,{l}_{p}$ according to the procedure set out in the previous paragraph. This requires information on ${v}_{{ji}}^{{\rm{EXIO}}}$ for $i={l}_{1},\ldots ,{l}_{p}$ and $j={r}_{1},\ldots ,{r}_{s}$. However, this detailed information is not available. Instead, we take ${v}_{{ri}}^{{\rm{EXIO}}}$. That is,

$$\begin{array}{cc}{\bar{v}}_{{ji}}^{{\rm{FIG}}-{\rm{e}}}={v}_{{jl}}^{{\rm{FIG}}}\frac{{v}_{{ri}}^{{\rm{EXIO}}}}{{\sum }_{i={l}_{1}}^{{l}_{p}}{v}_{{ri}}^{{\rm{EXIO}}}},\, & {\rm{for}}\,i={l}_{1},\ldots ,{l}_{p}\,{\rm{and}}\,j={r}_{1},\ldots ,{r}_{s}\end{array}$$

(the red part in Fig. 2b).

In the example of Fig. 2b, products CPA_Q86 and CPA_Q87_88 in FIGARO is in FIGARO-E3 kept into the same two products, but in the crosses with industry C10T12 in FIGARO, the initial split (before ulterior balancing processes) is done following the structure given by the 12 cited corresponding industries of EXIOBASE from column “A_HEAL” (the single product corresponding to the two cited products CPA_Q86 and CPA_Q87_88).

There are some exceptions that had to be dealt with while implementing this approach. For instance, there were cases in which a non-zero FIGARO cell to be disaggregated whilst the corresponding EXIOBASE cells were all zero. In other cases, some value added categories in FIGARO had no direct equivalence in EXIOBASE. The special cases and the solutions adopted are described in more detail in the SM (S2).

Following the construction of the initial FIGARO-E3 supply and use tables, several imbalances in the row and column totals between the supply and use remain. To deal with these, the same totals for products and industries are assumed (taking the average of the supply and use values) and the bi-proportional adjustment (GRAS) method^51–55^ has been used: once for the use table and once for the supply table. The GRAS-type adjustment used to balance tables separates the data into positive and negative matrices and iteratively adjusts row and column totals while preserving the sign of the original data. The method achieves convergence by applying ratios iteratively to maintain consistency with the FIGARO data aggregate constraints. The iterative procedure involves adjusting row and column totals using a GRAS balancing technique, followed by a second step that applies ratios to readjust the data based on aggregate totals from the original FIGARO data. This approach focuses on computational feasibility, avoiding the balancing of millions of data points with several constraints at once.

Hence, the row and column (equal for the supply and use table) constraints ensure that they are balanced in FIGARO-E3. Likewise, that aggregating the FIGARO-E3 tables preserves the FIGARO original aggregates as much as possible. In particular, with a perfect match for the Supply, and small discrepancies for the Use, being exactly 1 or around 1 most of the ratios between the estimates aggregated to the FIGARO level and the original FIGARO values, see the “Technical Validation” below, and with a total Weighted Absolute Percentage Error below 0.5, see “Quality assurance and Technical Validation” in the SM.

In the final step, the MRSUTs were transformed into industry-by-industry and product-by-product MRIOTs following the same procedure used originally in FIGARO^19^. Specifically, models B and D described in the Eurostat Manual of Supply, Use and Input-Output Tables^56^ were used. Model B uses the ‘industry technology assumption’, which assumes that each industry has a unique production process, independent from the product mix it produces. Model D, on the other hand, uses the ‘fixed product sales structure assumption’, thereby assuming that each product has its own specific sales structure, independent from the industry where it is produced.

### Labour accounts

The labour accounts consider total employment (persons), including the distinction of employment by gender and by skill. The aggregate country-specific data has been obtained from the OECD^43^. In absence of world totals that could be used to estimate employment in the ‘rest-of-world’ region, the data of the OECD has been complemented with data from EXIOBASE. The OECD data, which is given with a resolution of 36 industries, has been disaggregated to the FIGARO-E3 classification with EXIOBASE country employment data. Labour accounts are benchmarked with the OECD’s data. The process of labour accounts development is then independent from the compensation to employees (value added component) development, but a final constraint to avoid large outliers of wages (as ratio of compensation to employees to employment) and employment to output ratios is performed.

The split by gender and skill at 11 industry-level has been taken directly from the OECD^43^. In a first step, total employment by gender and skill is disaggregated to match the total employment data at 36 industry-level using EXIOBASE shares. The disaggregation to the 176 FIGARO-E3 industries is also done with EXIOBASE structures. Given that the OECD does not provide the mix of gender-skill combination, some mismatches arise when disaggregating the gender and skill data (3 skills × 2 gender per FIGARO-E3 industry *i*) separately. These are balanced through a bi-proportional adjustment procedure benchmarked to all the used OECD data. In the case of skills, we develop two versions, following the OECD definitions: of skill by education and of skill by occupation. The balancing above fully complies with OECD data at aggregate industry-levels, but do not ensure equal total labour by industries *i* between the two versions. We take an average sum by gender and impose strictly this new gender constraint in a final balancing.

### Energy accounts

The energy accounts produced comprise nine main indicators: primary energy supply, gross energy supply, gross energy use, net energy use, final energy use, energy industry own use, energy losses, non-energy use and emission relevant energy use. Primary energy supply, net energy use, final energy use, energy industry own use, energy losses and non-energy use are made available in the FIGARO-E3 database. The other three are intermediate products required to produce either the energy extensions made available with the database or the GHG emission accounts. The definitions of each indicator can be found in^57^ and in the SM.

The energy accounts are based on the IEA extended energy balances^44,45^. After processing the data and dealing with unbalances in trade data and some inconsistencies in electricity production efficiencies as explained in the SM of^41^, the IEA data is split in gross energy supply and gross energy use tables. These tables represent energy products and energy flows as documented by the IEA^58^. The IEA data, which follows the territory principle, is then aligned with the accounting principles of the (monetary) MRSUT, which follows the residence principle. The differences between these principles and their practical implications are described in^59^. The bridging procedure is undertaken with auxiliary transport data from various sources^59–61^ building on the approach described in^41,59^. Once the gross energy use and gross energy supply data is transformed into the territory principle, specific energy product/energy flow combinations are used to create emission-relevant energy use and net energy use tables^41^. Final energy use, energy industry own use and energy losses are subcomponents of net energy use.

To produce the energy extensions, the information given for each energy flow needs to be allocated to the FIGARO-E3 industry and final consumption classification. The allocation process builds on a correspondence table between energy flows and FIGARO-E3 industries and final consumption categories. Thus, the allocation of the energy data of each flow is based on the monetary proportions of the relevant intermediate and final consumption of energy products taken from the FIGARO-E3 MRSUT described above.

### GHG emission accounts

The GHG emission accounts in FIGARO-E3 include four main GHG categories: CO_2_, CH_4_, N_2_O and F-gases. GHG emissions comprise emissions from combustion and non-combustion processes. Combustion emissions are calculated by multiplying the emission-relevant energy use data tables obtained in an intermediate step of the energy extensions by relevant emission factors obtained from the Intergovernmental Panel for Climate Change^46^. The resulting emission tables, which are energy flow specific as defined by the IEA^58^, are then allocated to FIGARO-E3 industries based on the monetary proportions of intermediate and final consumption of relevant energy products taken from the FIGARO-E3 MRSUT. To that end, the same correspondence table referred to in the previous subsection is used.

Emissions from non-combustion processes cover fugitive emissions from fuels, industrial processes and product use, agriculture and waste. The data has been obtained from background data of the EDGAR database^47^ made available by the Joint Research Centre of the European Commission to build the FIGARO-E3 database. The database provides information on 117 different emission sources from non-combustion processes. The emissions have been allocated to the FIGARO-E3 classification based on a correspondence table between emission sources and the FIGARO-E3 industry and final consumption categories (see SM). In this case, the allocation of the emissions of each GHG category is done based on the monetary output of the relevant industries and final consumption categories according to the FIGARO-E3 MRIOT.

The sum of combustion and non-combustion emissions yields the GHG emission extension for non-European countries. In the case of European countries, the sum of both items has been rescaled to the official Eurostat GHG emission accounts, which are given at a 64 industry resolution^48^.

## Data Records

The datasets associated with this work^62–86^, can be found either on the Big Data Analysis Platform of the European Commission (JRC-BDAP) at https://jeodpp.jrc.ec.europa.eu/ftp/jrc-opendata/FIGARO-E3/, or at the Joint Research Centre Data Catalogue https://data.jrc.ec.europa.eu/collection/id-00403. A CC-BY licence applies.

The database contains a classification file^62^, a code file^63^, a metadata file^64^ and 22 data files (one of which contains the classifications used). The 22 data files are presented in two types of csv files, one in list format (eight files) and the other one in matrix format (14 files). All files include a specific identifier. The content of the files is shown in Table 1. In total, the list files take around 15 GB, while the matrix files take around one GB. In this context, the reader should note that the same data is provided in both types of files. To the extent possible, the format of all the files has been aligned with that of the original files from the FIGARO database. A metadata file is also available providing details about each of the building blocks of the FIGARO-E3 database.

**Table 1:** Main files of the FIGARO-E3 database.

Block	List file	Matrix file
MRSUT and MRIOT	Supply^65^	Supply^66^
	Use^67^	Use^68^
	Input-output industry by industry (industry-technology assumption)^69^	Input-output industry by industry (industry-technology assumption)^70^
	Input-output product by product (fixed product sales structure assumption)^71^	Input-output product by product (fixed product sales structure assumption)^72^
Labour	Employment by gender and skill^73^	Employment by gender and skill (occupation)^74^
		Employment by gender and skill (education)^75^
Energy	Energy^76^	Energy (primary energy supply)^77^
		Energy (net energy use)^78^
		Energy (final energy use)^79^
		Energy (energy industry own use)^86^
		Energy (energy losses)^80^
		Energy (non-energy use)^81^
GHG emissions	GHG emissions (in kt)^82^	GHG emissions (in kt)^83^
	GHG emissions (in kt CO2e)^84^	GHG emissions (in kt CO2e)^85^

## Technical Validation

A series of consistency checks have been undertaken to validate the different elements of the FIGARO-E3 database, particularly, in four main domains.

- MRIO database
- Labour accounts
- Energy accounts
- GHG emission accounts

### Multi-regional supply, use and input-output tables

For quality assurance, the deviations between the original FIGARO cells and the disaggregated FIGARO-E3 cells are compared after the GRAS balancing run. The focus has been set on ratios that are higher than two or lower than 0.5. The results are shown in Table 2. As shown in the table, there are 7,309 cases (0.18% of the 4.1 million of non-zero values in the original FIGARO MRIO database) with ratios above two, and 1,989 (0.05%) below 0.5. Most of the cases with ratios above two, namely 7,188 (98.34%) are associated with original cell values below 100 million €. In the case of ratios < 0.5, 23 instances (1.16%) referred to original values are greater than 100 million €, while there are no instances with original values lower than -100 million €.

**Table 2:** From the cases of ratios > 2 and ratios < 0.5, values of cells above and below original value.

Original value (Million €)	Ratios > 2		Ratios < 0.5	
	Number of cells	% of cases	Number of cells	% of cases
>100	121	1.66%	23	1.16%
>50	182	2.49%	32	1.61%
>0	7301	99.89%	1964	98.74%
<0	8	0.11%	25	1.26%
<−50	0	0.00%	2	0.10%
<−100	0	0.00%	0	0.00%
Total	**7309**	**100.00%**	**1989**	**100.00%**

The above was just referred to what we may consider “too high” or “too low” ratios. The SM provides additional insights and material on these validations. E.g. Table 3 in the SM illustrates, for those absolute ratios above 2 (for the 7309 cases), the largest original values (ranked by them) and the exact ratios.

**Table 3:** Number and % of Aggregate FIGARO-E3 to FIGARO ratios (cases) for all the non-zero data points.

Condition of ratio	Number of cells	% of cases	Condition of ratio	Number of cells	% of cases
= > 1	1957050	47.6%	<1	2152677	52.4%
>1,01	205166	5.0%	<0,99	274032	6.7%
>1,05	37750	0.9%	<0,95	66259	1.6%
>1,1	23833	0.6%	<0,9	30911	0.8%
>1,15	18964	0.5%	<0,85	19328	0.5%
>1,25	15605	0.4%	<0,75	10144	0.2%
>1,5	9281	0.2%	<0,5	1989	0.05%
>2	7309	0.2%	<0,25	63	0.002%
>5	1825	0.04%	<0,15	7	0.000%
>10	1755	0.04%	<0,05	4	0.000%

Table 3 provides the results for all the (more than 9 million cells, 4.1 million of non-zero) data point ratios. The nice thing of those (AGG_FIG-E3 to FIG) ratios is that all provide insights in relative terms, because they all compare to the original FIGARO value. We may observe looking at the difference between rows 1 and 2 of figures, that 43% of all ratios fall within 1 and 1.01, and 46% between 1 and 0.99. Then looking at the difference between rows 2 and 3 we see that more than 4% fall between 1.01 and 1.05 and more than 5% between 0.99 and 0.95. The share of non-zero ratios (and hence data points) which fall outside the 5% deviation is then 0.9% (above 1.05) and 1.6% (below 0.95). Outside the 15% deviation is less than 0.5% of cases in both directions.

Beyond the checks undertaken to test the consistency of the disaggregation process, we have also compared the differences between FIGARO-E3 and other MRIO databases, on the one hand, and FIGARO, on the other, to understand how the former deviate from the latter, which, as argued before, can be considered official statistical. Thus, relative differences have been computed through the Weighted Average Percentage Error (WAPE). Here, WAPE is calculated by taking the observed values and the official values, and calculating the error between those two values. The formulation is further explained in the SM. Thus, the WAPE between FIGARO and FIGARO-E3 (symmetric table type D) is 1%, while WAPE between FIGARO and the OECD Inter-Country Input-Output tables^18^, GLORIA^87^ and EXIOBASE^41^ is 34%, 39% and 83% respectively. The biggest differences are found in the IO tables of the ‘rest-of-world’ region, China, US and Japan as shown in the SM.

### Labour accounts

As described above, the disaggregation of the labour figures obtained from the OECD^43^, has been done respecting the original values. Thus, there is first a match between totals from the OECD at 36-industry level for the aggregation of the FIGARO-E3 industries at the same 36-industry resolution. Then for the 11-industry level for skill and gender and the aggregation of the FIGARO-E3 accounts at the same 11-industry resolution.

### Energy accounts

For the energy accounts, two main checks have been undertaken to ensure that there are no errors when creating the energy tables and when allocating the data to industries and final consumption categories. At the global level, primary energy supply needs to match net energy use. The division of the latter by the former equals 100.00%. At the country level, gross energy supply needs to match gross energy use. This is the case for every country.

### GHG emission accounts

For GHG emissions, the main consistency check undertaken refers to the consistency between the emission extension and the Eurostat emission accounts after the reconciliation step. Thus, full consistency is achieved between the emission extension of the European countries and the Eurostat data at 64 industry level.

## Usage Notes

The files provided can be used to calculate carbon and labour footprint indicators using the standard input-output equations. Likewise, they can be used to analyse global supply chains or to build multi-sector, multi-country partial and general equilibrium models.

The resulting footprint indicators are expected to be more accurate than those using databases that are not consistent with official statistics. Nonetheless, care should be taken when interpreting the results of individual products and industries. In this vein, energy and GHG footprints are expected to be more robust for aggregate consumption categories, given that the disaggregation process introduces additional uncertainty. Likewise, labour footprints are expected to be more robust when presented at the original 36-industry grouping, while in the case of gender and skill, the results will be more reliable at the original 11-industry level. Nevertheless, it is worth noting that aggregate footprints are expected to be more reliable using FIGARO-E3 than official FIGARO, since auxiliary data has been used for allocating environmental data to industries, which reduces potential aggregation biases.

For energy footprints, the use of primary energy supply and net energy use is recommended to avoid double accounting^57^. For this reason, only these two extensions have been made available.

This version of the database is provided for the year 2015. Time series may be generated in future releases.

## Supplementary information

Supplementary Material

## Data Availability

The GAMS code files^63^ have been included in the database: http://data.europa.eu/89h/4cfa6fac-d622-4f9a-8eec-218acb657509.
