# Supplementary material for: FIGARO-E3: a high-resolution extended multi-regional input-output database consistent with official statistics
Source: Sci Data. 2025 Apr 4;12:575. doi: 10.1038/s41597-025-04431-z (PMC11971246; doi:10.1038/s41597-025-04431-z)
Supplement: Supplementary file 1 — Supplementary Material [file 41597_2025_4431_MOESM1_ESM.docx]

### Supplementary material

### Computing FIGARO-E3 Inter-country Supply, Use and Input-Output Tables

### Goal

This section explains how the original FIGARO tables were disaggregated to fit the classification of FIGARO-E3 (in the Appendix of the article), based on the structures of EXIOBASE v3.8.2 (Stadler et al., 2018b).

We depart from $z_{kl}^{\mathrm{FIG}}$ and $z_{ji}^{\mathrm{EXIO}}$, which represent each of the generic (without distinction whether it belongs to the Use or Supply table for simplicity) elements of the matrices in FIGARO and EXIOBASE respectively.

In general, we may say that several products of $j$ correspond to a specific product of $k$, say of $q$ elements (and the same for industries of $i$ correspond to a product of $l$, say of $p$ elements), although that is not always the case. In some cases, such as especially for some services, we find the opposite. $u_{kl}^{\mathrm{FIG}}$ and $u_{ji}^{\mathrm{EXIO}}$ represent each of the elements of the matrices in FIGARO and EXIOBASE respectively of the use table. $v_{kl}^{\mathrm{FIG}}$ and $v_{ji}^{\mathrm{EXIO}}$ represent each of the elements of the matrices in FIGARO and EXIOBASE respectively of the supply table.

$\bar{z}_{ji}^{FIG-e}$ represents each of the generic elements of the matrices in FIGARO-E3, with products $j$ and industries $i$.

### Initial split

In the first step, we provide an initial estimate with two basic cases. The first case is where one industry and/or product in FIGARO corresponds to several industries/products in EXIOBASE. In that case, FIGARO-E3 disaggregates the FIGARO data using the shares from EXIOBASE. Assume that industry $l$ in FIGARO corresponds to industries $i=l_{1},\ldots,l_{p}$ in EXIOBASE and/or that product $k$ in FIGARO corresponds to products $j=k_{1},\ldots,k_{q}$. In that case, we have

$\bar{v}_{ji}^{FIG-e}=v_{kl}^{\mathrm{FIG}}\frac{v_{ji}^{\mathrm{EXIO}}}{\sum_{i=l_{1}}^{l_{p}} \sum_{j=k_{1}}^{k_{q}} v_{ji}^{\mathrm{EXIO}}}$, for $i=l_{1},\ldots,l_{p}$ and $j=k_{1},\ldots,k_{q}$ (the red part in Figure 1a) (1)

$\bar{v}_{ji}^{FIG-e}=v_{kl}^{\mathrm{FIG}}\frac{v_{ij}^{\mathrm{EXIO}}}{\sum_{j=k_{1}}^{k_{q}} v_{ji}^{\mathrm{EXIO}}}$, for $i\neq l_{1},\ldots,l_{p}$ and $j=k_{1},\ldots,k_{q}$ (the green part in Figure 1a) (2)

$\bar{v}_{ji}^{FIG-e}=v_{kl}^{\mathrm{FIG}}\frac{v_{ji}^{\mathrm{EXIO}}}{\sum_{i=l_{1}}^{l_{p}} v_{ji}^{\mathrm{EXIO}}}$, for $i=l_{1},\ldots,l_{p}$ and $j\neq k_{1},\ldots,k_{q}$ (the blue part in Figure 1a) (3)

Here, $v_{ji}^{\mathrm{EXIO}}$ gives the supply of product $j$ by industry $i$ in EXIOBASE, $\bar{v}_{ji}^{FIG-e}$ gives the estimate (indicated by an overbar) in FIGARO-E3, and $v_{kl}^{\mathrm{FIG}}$ gives the aggregated supply in FIGARO. The same procedure is used to estimate the use of product $j$ in industry $i$ (i.e., $\bar{u}_{ji}^{FIG-e}$). In the example of Figure 2a, industry C10T12 in FIGARO is split into 12 industries in FIGARO-E3 (C10_A, ..., C10_J, C11, C12) following the EXIOBASE classification. Also, product CPA_B in FIGARO is in FIGARO-E3 split into 23 products (CPA_B05_A, ..., CPA_B05_H, CPA_B06_A, ..., CPA_B06_D, CPA_B07_A, ..., CPA_B07_H, CPA_B08_A, ..., CPA_B08_C) following the EXIOBASE classification.

The second case is where several industries and/or products in FIGARO correspond to one industry/product in EXIOBASE. In that case, we adopt the detailed information from FIGARO and where necessary, we take the shares for the aggregated EXIOBASE industries/products in the process of disaggregation. Assume that the aggregate product $r$ in EXIOBASE corresponds to products $j=r_{1},\ldots,r_{s}$ in FIGARO (see the yellow columns in Figure 1b). The industry $l$ in FIGARO, however, needs to be disaggregated into industries $l_{1},\ldots,l_{p}$ according to the procedure set out in the previous paragraph. This requires information on $v_{ji}^{\mathrm{EXIO}}$ for $i=l_{1},\ldots,l_{p}$ and $j=r_{1},\ldots,r_{s}$. However, this detailed information is not available. Instead, we take $v_{ri}^{\mathrm{EXIO}}$. That is,

$\bar{v}_{ji}^{FIG-e}=v_{jl}^{\mathrm{FIG}}\frac{v_{ri}^{\mathrm{EXIO}}}{\sum_{i=l_{1}}^{l_{p}} v_{ri}^{\mathrm{EXIO}}}$, for $i=l_{1},\ldots,l_{p}$ and $j=r_{1},\ldots,r_{s}$ (the red part in Figure 1b).

In the example of Figure 1b, products CPA_Q86 and CPA_Q87_88 in FIGARO is in FIGARO-E3 kept into the same two products, but in the crosses with industry C10T12 in FIGARO, the initial split (before ulterior balancing processes) is done following the structure given by the 12 cited corresponding industries of EXIOBASE from column “A_HEAL” (the single product corresponding to the two cited products CPA_Q86 and CPA_Q87_88).

Figure 1: Graphical representation of the initial split of FIGARO Supply (in the example) and Use tables.


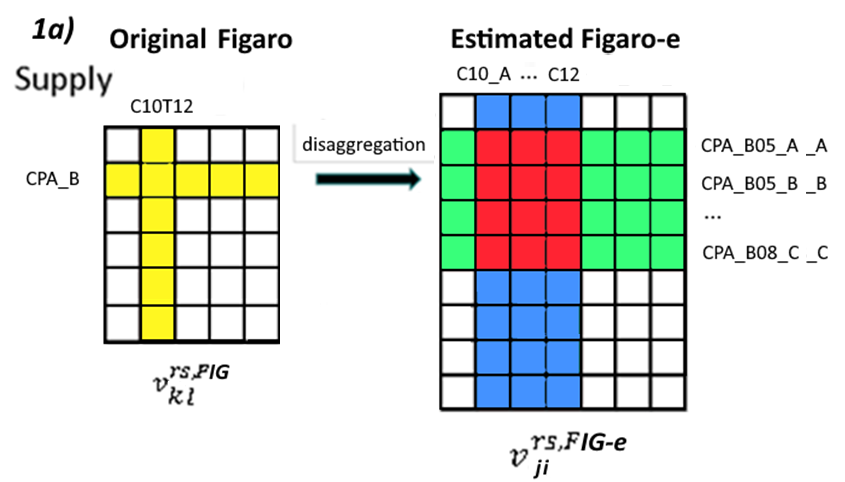

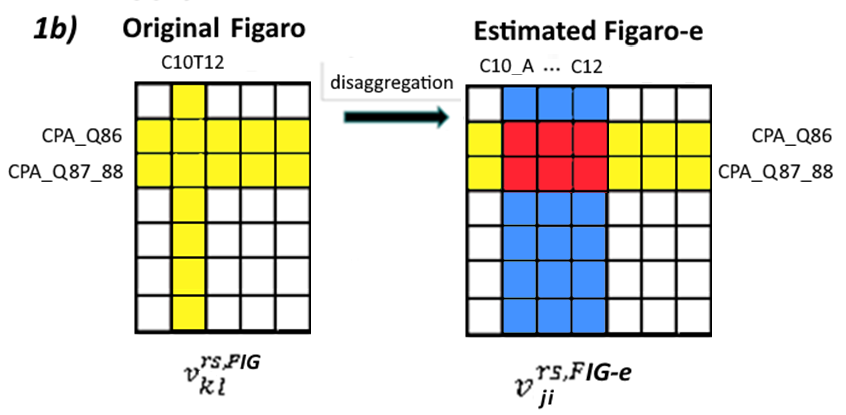


Source: own elaboration

| **Empirical challenges:**  In empirical applications, simple formulae described as the above often encounter data challenges. At this point, we may highlight the following challenges and the adopted solution:   1. a) Non-zero values in the Agg_FIG and zero in the Agg_EXIO: We distribute the original Agg_FIG with the general structures (shares) of the country.   b) Lacking EXIOBASE structures for the product-industries combination: We use general (average) structures for all countries taken together (an alternative would be to take structures to “the closest” country under some metric, but at some structures for specific data points, e.g. cells of final demand such as NPSH or changes in inventories and valuables of certain products lacks for several countries).   1. Lack of detailed structure for the Supply “P7, Imports CIF”, “D21X31, Taxes less subsidies on products” and “OTTM- Trade and transport margins” (lack of such values in EXIOBASE): We use that of the total (“P1_TC, Total output by product”). 2. For the Use, lack of “C07 Taxes less subsidies on products” structure: We use that of “B1G” “Gross value added at basic prices”. 3. “OTH” (CIFFOB, C02, C05) does not have an initial structure in EXIOBASE: the original Figaro values are introduced. |
| --- |

### Common Supply and Use totals

For the method of construction, these initial estimates will imply that $\sum_{r} \sum_{j} \bar{u}_{ji}^{rs,FIG-e}\neq\sum_{r} \sum_{j} \bar{v}_{ji}^{rs,FIG-e}$ and $\sum_{s} \sum_{i} \bar{u}_{ji}^{rs,FIG-e}\neq\sum_{s} \sum_{i} \bar{v}_{ji}^{rs,FIG-e}$, but these are constraints that must hold. Accordingly, we obtain:

$\bar{u}_{i}^{s,FIG-e}=\bar{v}_{i}^{s,FIG-e}=\frac{\sum_{r} \sum_{j} \bar{u}_{ji}^{rs,FIG-e}+\sum_{r} \sum_{j} \bar{v}_{ji}^{rs,FIG-e}}{2}$ (1)

$\bar{u}_{j}^{s,FIG-e}=\bar{v}_{j}^{s,FIG-e}=\frac{\sum_{r} \sum_{i} \bar{u}_{ji}^{rs,FIG-e}+\sum_{r} \sum_{i} \bar{v}_{ji}^{rs,FIG-e}}{2}$ (2)

Being $\bar{u}_{i}^{s,FIG-e}$ and $\bar{u}_{j}^{s,FIG-e}$ obviously the elements of the average objective values searched, of the row and vector totals representing the common product-country and industry-country accounts. In other words, the searched totals of the Use are equal to those of the Supply for the product-country and industry-country, to which in the Use the Final demand totals and “VA” type (“B1G”, “C07” and “OTH”) totals.

| **Empirical challenges:**   1. Essentially the main challenge at this point is that, based on the EXIOBASE structures for disaggregation, but also being applied to the FIGARO aggregates (which may also have some zeroes where there were none in EXIOBASE) is that a (row or column) total in the Supply is zero and non-zero in the Use table (or vice versa, although we do not obtain any case as such). Two options are possible at this point: a) to create an average value anyway, introducing arbitrary (before balancing) non-zero elements in the other table; b) to make that (row or column) total zero, which is our preferred option. 2. A second potential error is obtaining a positive value in one of the tables (Supply or Use) and a negative in the other. In the event of such rare case, we still would adopt the average value (found in marginal cases for previous versions of the FIGARO and EXIOBASE data, resulting in small positive searched values). |
| --- |

Accordingly, the above ($\bar{u}_{i}^{s,FIG-e}=\bar{v}_{i}^{s,FIG-e}$ and $\bar{u}_{j}^{s,FIG-e}=\bar{v}_{j}^{s,FIG-e})$ are the constraints that are applied in two RAS-type method procedures (one for the Use table, and one for the Supply table), together with the fact (accounted already in the split) that $\sum_{i=l_{1}}^{l_{p}} \sum_{j=k_{1}}^{k_{q}} \bar{u}_{ji}^{FIG-e}=u_{kl}^{\mathrm{FIG}}$ and $\sum_{i=l_{1}}^{l_{p}} \sum_{j=k_{1}}^{k_{q}} \bar{v}_{ji}^{FIG-e}=v_{kl}^{\mathrm{FIG}}$, i.e., that when aggregating, the FIGARO original aggregates are preserved. This aggregation can be also defined for $\mathbf{U}^{FIG-e}=\{u_{kl}^{rs,FIG-e}\}$ and $\mathbf{V}^{FIG-e}=\{v_{kl}^{rs,FIG-e}\}$.

We also make use of the aggregation of the estimated data (initially with the first split, and in the following rounds) at the classification of FIG-e into the original FIGARO classification: $\sum_{i=l_{1}}^{l_{p}} \sum_{j=k_{1}}^{k_{q}} \bar{u}_{ji}^{FIG-e}=\tilde{u}_{kl}^{FIG-e}$ and $\sum_{i=l_{1}}^{l_{p}} \sum_{j=k_{1}}^{k_{q}} \bar{v}_{ij}^{FIG-e}=\tilde{v}_{lk}^{FIG-e}$

The two RAS-type procedures could be performed in several ways.

### (G,K,)RAS-type procedures options and choice

We talk about (G)RAS-type method but the first thing that becomes clear is that, since there are negative values in the Use table (e.g. in the final demand, in the “P5M” column of changes in inventories and valuables, or in “OTH” row) we treat appropriately these by separating the **P** and **N** matrices with a GRAS-type method. At some point, we also considered the possibility of allowing for sign flipping, but we finally discarded it, since in the end, lacking any further knowledge, we rather preserve the data points that are positive in the original FIGARO as positive and the negative as negative.

1^st^) The two GRAS-type procedures could be performed separately, just considering the Supply and Use described totals. This would lead to the common totals but, then, the internal consistency, i.e., the fact that FIGARO-E3 product-country and industry-country aggregations to the initial common FIGARO accounts, would not be ensured. As we explain in options three and four, this implies that also the original FIGARO-E3 product-country and industry-country aggregations (Agg_FIG) need to be ensured again.

2^nd^) A second potential option would be to perform it iteratively for every country in its combination of $i=l_{1},\ldots,l_{p}$ and its analogous $j=r_{1},\ldots,r_{s}$, that is, for every original FIGARO product and analogous/corresponding industry that is disaggregated in each country. In Figure 1, the table would balance the values of the coloured columns and rows, and make zero all the other elements in the table. The logic would be that if applying a GRAS-type method only to such combination, one can ensure the original aggregate with such constraint and at the same time adapting the total values. The problem of this method is that this works for the first block, but then when being applied iteratively, the already balanced combinations could deviate.

3^rd^) As indicated in the first option, after a GRAS balancing of the totals (of the Supply and Use tables) the original FIGARO-E3 product-country and industry-country aggregations (Agg_FIG) need to be ensured again. A third option then, conceptually the most elegant, would be to perform the balancing on both the totals and the internal aggregations needed. In order to do so, one could make a system such as the one (multidimensional nD-GRAS) described in Valderas-Jaramillo & Rueda-Cantuche (2021) or in (Lenzen et al., 2009), i.e., a system of $\mathbf{Ga=c}$ where $\mathbf{a}$ is the vectorisation of the table at hand ($\bar{U}^{FIG-e}$ or $\bar{V}^{FIG-e}$), $\mathbf{c}$ are the constraints (in this case, even once excluded the non-zero values, for example for the Use, 8,244 of the column totals, 8,240 of the row totals and 5,660,416 original FIGARO datapoints or Agg_FIG to preserve) and $\mathbf{G}$ the matrix of zeros and ones representing the vectorized constraints, with the dimension of $\mathbf{c}$ in rows times the number of columns of the vectorized table (in the example of the Use, 8,244x8,240). In other words, a 5,676,900x6,793,0560 (array) matrix. Computationally, this operation is not easily viable in a regular computer. For example, in this form many runs are needed to obtain both for the rows and column balancing (performed in two steps in a traditional GRAS to obtain the so-called r and s, while needing 8,244+8,240 resolutions to obtain the unique r in the KRAS) and specially to obtain the other 5,660,416 data points (while simple ratios with correspondence matrices can be applied to ensure the Agg_FIG again). Given some of these and other issues, there are other solutions that have been discussed in the literature with similar logics, for example de la Torre Cuevas et al. (2023).

4^th^) Given the above, looking for some pragmatism given our computation capacities, and without losing that logic of point 3 of imposing all constraints in some iterative form, the following is the option we implemented, which is an iterative balancing of the totals (with a GRAS method) and a “balancing” (application of the ratios) of the Agg_FIG. In this second balancing (the application of the ratios), $\bar{u}_{ji}^{FIG-e}$ and $\bar{v}_{ij}^{FIG-e}$ get readjusted in the following form:

$\bar{u}_{ji}^{FIG-e}=\bar{u}_{ji}^{FIG-e} \frac{u_{kl}^{\mathrm{FIG}}}{\tilde{u}_{kl}^{FIG-e}}$ ; $\bar{v}_{ji}^{FIG-e}=\bar{v}_{ji}^{FIG-e} \frac{v_{kl}^{\mathrm{FIG}}}{\tilde{v}_{kl}^{FIG-e}}$ (6)

Where as indicated the ratio is composed by the original FIGARO value to current sum ratio. The current sum (at each iteration) of FIG-e: $\sum_{i=l_{1}}^{l_{p}} \sum_{j=k_{1}}^{k_{q}} \bar{u}_{ji}^{FIG-e}=\tilde{u}_{kl}^{FIG-e}$ and $\sum_{i=l_{1}}^{l_{p}} \sum_{j=k_{1}}^{k_{q}} \bar{v}_{ji}^{FIG-e}=\tilde{v}_{kl}^{FIG-e}$

The iterative balancing considers the insights of the third option, but instead of balancing the vector (vectorized matrix) with millions of data points step by step, we update the block of row and column totals (GRAS), and then the block of aggregate (original FIGARO data) constraints by applying the ratio (e.g. for the Use table, $\frac{u_{kl}^{\mathrm{FIG}}}{\tilde{u}_{kl}^{FIG-e}}\mathbf{)}$. We call “an iteration” (obtaining the corresponding WAPE) after having applied both (the GRAS and the ratios application). We acknowledge that the approach in Lenzen et al. (2009) is superior, especially conceptually, and convergence is achieved likely more smoothly (every iteration ensures converging to the corresponding constraint of $\mathbf{c}$**,** without pushing all the way the solution towards certain constraints while ignoring others), but computationally, for this case, we reach convergence in a faster (actually, only feasibly) in this way.

Another important point is that due to the much lower degrees of freedom (much more zero data points) of the Supply table, we first perform the balancing of the Supply table, so that in case of lacking the full achievement of the searched totals constraints (while matching the original FIGARO Agg_FIG), then the common Use searched totals can be imposed equal to those of the Supply (deviating slightly from the average estimated in equation 16). We stop the algorithm then at a point of matching the original FIGARO Agg_FIG for the Supply ($\tilde{v}_{lk}^{FIG-e}=v_{lk}^{\mathrm{FIG}} )$. Then, for the Use table, we stop the algorithm at a point of matching the Supply row and column common totals.

Furthermore, with the approach taken, we may see at all points the convergence to the totals (as in any GRAS) and the described ratios, $\frac{v_{kl}^{\mathrm{FIG}}}{\tilde{v}_{kl}^{FIG-e}}$ and $\frac{u_{kl}^{\mathrm{FIG}}}{\tilde{u}_{kl}^{FIG-e}}$. By construction (updating first the Supply), we have already indicated that $\frac{v_{kl}^{\mathrm{FIG}}}{\tilde{v}_{kl}^{FIG-e}}$ =1 for all cases, so that we perfectly match the FIGARO table. However, for the Use table, since we stop the algorithm at the GRAS balancing of the total rows and columns, we do not necessarily get all $\frac{u_{kl}^{\mathrm{FIG}}}{\tilde{u}_{kl}^{FIG-e}}=1$. Both the GRAS and the ratio were applied 20 times.

MRIO table

In order to compute the (typically called symmetric) IO table/s, we follow the original FIGARO (Rémond-Tiedrez & Rueda-Cantuche, 2019)and compute Transformations that do not yield negatives (i.e. Model B and Model D, see Eurostat, 2008).

### Quality assurance and Technical Validation

For quality assurance, we compute a few indicators that compare the deviations from the original FIGARO Agg_FIG after each GRAS balancing run. The ratios are obtained in files (“u_Ratios_FIG_toFIG_E_Use_%run number%.gdx), representing $\frac{u_{kl}^{\mathrm{FIG}}}{\tilde{u}_{kl}^{FIG-e}}$ for every “Agg_FIG” element (original FIGARO data, variable “FIG_to_FIG_E_est_Use” in the gdx files) and for every FIGARO-E3 element (variable “FIG_to_FIG_E_est_Use_fig_e”, where all disaggregated FIGARO-E3 elements obviously show the same ratio than the “Agg_FIG element”). There are a few large (in absolute terms, although negative ratios are rare) minimum and maximum values of these ratios.

In particular, we compute the marginal values for the cases larger (in absolute terms) than $\frac{u_{kl}^{\mathrm{FIG}}}{\tilde{u}_{kl}^{FIG-e}}>\delta$ and smaller (in absolute terms) than $\frac{u_{kl}^{\mathrm{FIG}}}{\tilde{u}_{kl}^{FIG-e}}<\delta$.

To have the intuition of the numbers, it must be noted that we compare the 5,658,133 non-zero data points of the original Figaro Use table (“FIG_MR_Use”) with the aggregated final (balanced) table from FIGARO-E3. The total value of the Use is ~195 trillion euro, and the average value excluding zeros is ~35 (~19 including zeroes). Of those outliers/cases, we also obtain the value itself (again $\bar{u}_{ji}^{FIG-e}=\bar{u}_{ji}^{FIG-e} \frac{u_{kl}^{\mathrm{FIG}}}{\tilde{u}_{kl}^{FIG-e}}$ if we look at the FIGARO-E3 values) and the original value (in “FIG_MR_Use”) $u_{kl}^{\mathrm{FIG}}$, in million euro.

In particular, we find 7,309 cases (that is 0.18% of the 4,107,904 of non-zero values in the original FIGARO and hence potential ratios) of ratios above 2 (i.e. $\delta=2)$, and 1,989 (0.05%) below 0.5 $(\delta=0.5)$. We try to verify that most of all those cases are due to very marginal values. Most of them (7,188, 98%) are values of cells below 100 million euro. Or, inversely, as shown in Table 1, only 121 (1.66%) are above 100 million euro. Similarly, 182 (2.49%) are above 50, still 567 (7.76%) above 5 and 1,171 (16%) above one and 2,632 (36%) above 0.1.

Table 1: From the cases of ratios >2 ($\boldsymbol{\delta}\mathbf{=2)}$ and ratios <0.5 ($\boldsymbol{\delta}\mathbf{=0.5)}$ respectively, values of cells above and below X Million euro

|  | **Too high ratios** | | | | **Too low ratios** | | | |
| --- | --- | --- | --- | --- | --- | --- | --- | --- |
| Actual obtained value (million euro): | Number of cells | % of  “too high” cases | % of all non-zero values | Number of cells | | % of “too low” cases | % of all non-zero values |  |
| >10,000 | 5 | 0.07% | 0.000122% | 0 | | 0.00% | 0.000000% |  |
| >5,000 | 11 | 0.15% | 0.000268% | | 0 | 0.00% | 0.000000% |  |
| >2,000 | 25 | 0.34% | 0.000609% | 0 | | 0.00% | 0.000000% |  |
| >500 | 57 | 0.78% | 0.001388% | 3 | | 0.15% | 0.000073% |  |
| >100 | 121 | 1.66% | 0.002946% | 23 | | 1.16% | 0.000560% |  |
| >50 | 182 | 2.49% | 0.004430% | 32 | | 1.61% | 0.000779% |  |
| >20 | 294 | 4.02% | 0.007157% | 41 | | 2.06% | 0.000998% |  |
| >10 | 406 | 5.55% | 0.009883% | 49 | | 2.46% | 0.001193% |  |
| >5 | 567 | 7.76% | 0.013803% | 59 | | 2.97% | 0.001436% |  |
| >1 | 1171 | 16.02% | 0.028506% | 136 | | 6.84% | 0.003311% |  |
| >0.1 | 2632 | 36.01% | 0.064072% | 430 | | 21.62% | 0.010468% |  |
| >0 | 7301 | 99.89% | 0.177731% | 1964 | | 98.74% | 0.047810% |  |
| <0 | 8 | 0.11% | 0.000195% | 25 | | 1.26% | 0.000609% |  |
| <-1 | 1 | 0.01% | 0.000024% | 3 | | 0.15% | 0.000073% |  |
| <-10 | 1 | 0.01% | 0.000024% | 2 | | 0.10% | 0.000049% |  |
| <-50 | 0 | 0.00% | 0.000000% | 0 | | 0.00% | 0.000000% |  |
| **Total** | **7309** | **100.00%** | **0.177925%** | **1989** | | **100.00%** | **0.048419%** |  |

Source: own elaboration

We may also repeat the exercise for even closer to 1 ratios, i.e. “too high ratios” defined as $\delta$ =1.5 and “too low ratios” defined as $\delta=0.75$ as shown in Table 2.

Table 2: From the cases of ratios > $\mathbf{1.5}$ ($\boldsymbol{\delta}\mathbf{=1.5)}$ and ratios <0.75 ($\boldsymbol{\delta}\mathbf{=0.75)}$ respectively, max ratios and relation to values.

|  | **Too high ratios** | | | | **Too low ratios** | | | |
| --- | --- | --- | --- | --- | --- | --- | --- | --- |
| Actual obtained value (million euro): | Number of cells | % of “too high” cases | % of all non-zero values | Number of cells | | % of “too low” cases | % of all non-zero values |  |
| >10000 | 7 | 0.08% | 0.000170% | 0 | | 0.00% | 0.000000% |  |
| >5000 | 13 | 0.14% | 0.000316% | 0 | | 0.00% | 0.000000% |  |
| >2000 | 31 | 0.33% | 0.000755% | 1 | | 0.01% | 0.000024% |  |
| >500 | 73 | 0.79% | 0.001777% | 16 | | 0.16% | 0.000389% |  |
| >100 | 171 | 1.84% | 0.004163% | 93 | | 0.92% | 0.002264% |  |
| >50 | 257 | 2.77% | 0.006256% | 136 | | 1.34% | 0.003311% |  |
| >20 | 391 | 4.21% | 0.009518% | 233 | | 2.30% | 0.005672% |  |
| >10 | 526 | 5.67% | 0.012805% | 361 | | 3.56% | 0.008788% |  |
| >5 | 728 | 7.84% | 0.017722% | 553 | | 5.45% | 0.013462% |  |
| >1 | 1530 | 16.49% | 0.037245% | 1251 | | 12.33% | 0.030453% |  |
| >0.1 | 3388 | 36.50% | 0.082475% | 2898 | | 28.57% | 0.070547% |  |
| >0 | 9267 | 99.85% | 0.225589% | 10073 | | 99.30% | 0.245210% |  |
| <0 | 14 | 0.15% | 0.000341% | 71 | | 0.70% | 0.001728% |  |
| <-1 | 3 | 0.03% | 0.000073% | 13 | | 0.13% | 0.000316% |  |
| <-10 | 2 | 0.02% | 0.000049% | 6 | | 0.06% | 0.000146% |  |
| <-50 | 0 | 0.00% | 0.000000% | 3 | | 0.03% | 0.000073% |  |
| **Total** | **9281** | **100.00%** | **0.225930%** | **10144** | | **100.00%** | **0.246939%** |  |

Source: own elaboration

As shown in Table 1, the cases of “too low ratios” is even less worrying (in part this is due to the fact that we are just capturing ratios of absolute value 0-0.5, being the largest ones in the “too high ratios”). Out of the 1,989 cases, 1,853 (93%) of them are values below one million euro, or inversely seen as in Table 1, only 136 (7%) are above one. Some (3, i.e. 0.15%) are below minus one, being only two values below minus ten million euro and none below -30. This shows that the cases of “too low ratios” occur basically for marginal values. We obviously see even more clearly the exceptionality of those cases looking at the share that they represent with respect to the total number of non-zero values (the second column of %s in both cases in Table 1).

As an additional metric, we may also rank the largest ratios and see their values. Table 3 shows the ranking of the highest (and lowest, most negative) values in the original Figaro Use table from too high ratios (abs>2). Despite some commonalities, such as the frequency of cases in rows of large economies e.g. of US and China (CN) related to columns of the same type of countries of G47, o F, etc., we have not detected particular errors in the computation, but just differences derived from the differential initial structures between FIGARO and EXIOBASE, which later on get solved when applying the ratios, but the GRAS balancing drives them to the discrepancies found in the initial spilt. Some of those discrepancies are probably not entirely reconcilable. Still the analysis performed shows how after every run these cases are slowly decreasing both in number and in size. The way to read the table then is that 16180.52 million Euro is the cell with the highest value that has an actual aggregated FIG-E to original FIG absolute ratio above two. Of all ratios above two, the average absolute original value is 18.57.

Table 3: Ranking of the 1st highest absolute values in the original FIG_MR_Use from too high ratios (abs>2)

| **FIG_r_ctry** | **FIG_r_i** | **FIG_c_ctry** | **FIG_c_i** | **Actual Ratio**  **(abs ratio > 2)** | **Original Value, million Euro** |
| --- | --- | --- | --- | --- | --- |
| US | CPA_K64 | US | G47 | 2.838697 | 20228.8 |
| CN | CPA_L | CN | G47 | 3.481077 | 16180.52 |
| CN | CPA_K64 | CN | G47 | 3.481537 | 13815.85 |
| US | CPA_J62_63 | US | G47 | 2.830952 | 12618.31 |
| US | CPA_N77 | US | G47 | 2.81986 | 10782.07 |
| GB | CPA_C20 | GB | P3_S14 | 2.387603 | 9691.83 |
| CA | CPA_C25 | CA | F | 136.9456 | 7017.328 |
| US | CPA_P85 | US | G47 | 2.841851 | 6105.377 |
| CN | CPA_C13T15 | CN | G47 | 3.509119 | 5274.528 |
| US | CPA_I | US | G47 | 2.858456 | 5159.235 |
| US | CPA_C33 | US | G47 | 2.720443 | 5067.317 |
| US | CPA_C22 | US | G47 | 2.834539 | 4238.131 |
| RU | CPA_B | RU | P51G | 2.37694 | 4231.797 |
| US | CPA_C10T12 | US | G47 | 2.860481 | 4135.261 |
| US | CPA_F | US | G47 | 2.862721 | 4009.018 |
| CN | CPA_I | CN | G47 | 3.507936 | 3811.731 |
| IN | CPA_D35 | IN | O84 | 4.334481 | 3776.742 |
| ID | CPA_B | ID | D35 | 2.667001 | 3757.611 |
| US | CPA_C18 | US | G47 | 2.766087 | 3573.823 |
| CN | CPA_C18 | CN | G47 | 3.449796 | 3433.379 |
| IN | CPA_C19 | IN | O84 | 80.24901 | 3173.564 |
| US | CPA_K66 | US | G47 | 2.849134 | 3130.031 |
| CN | CPA_J61 | CN | G47 | 3.503288 | 2770.637 |
| US | CPA_C17 | US | G47 | 2.845952 | 2179.947 |
| CN | CPA_C17 | CN | G47 | 3.507281 | 2159.238 |
| AU | CPA_K64 | AU | G47 | 3.482323 | 1717.271 |
| US | CPA_C25 | CA | F | 189.2784 | 1596.602 |
| CN | CPA_H52 | CN | G47 | 3.499694 | 1594.24 |
| CA | CPA_C20 | CA | C22 | 4.471311 | 1467.041 |
| CN | CPA_S96 | CN | G47 | 3.504079 | 1260.462 |
| US | CPA_C16 | US | G47 | 2.847048 | 1236.476 |
| CN | CPA_H53 | CN | G47 | 3.443765 | 1070.715 |
| AU | CPA_F | AU | G47 | 3.497848 | 1016.938 |
| CN | CPA_C25 | CA | F | 190.7557 | 990.641 |
| RO | CPA_C29 | RO | H49 | 2.467052 | 947.444 |
| US | CPA_A03 | CA | C10T12 | 2.465997 | 922.696 |
| US | CPA_C26 | US | G47 | 2.868093 | 870.475 |
| US | CPA_C31_32 | US | G47 | 2.863049 | 855.433 |
| US | CPA_C25 | US | G47 | 2.852267 | 845.284 |
| US | CPA_K64 | CA | F | 188.3778 | 837.918 |
| US | CPA_J58 | US | G47 | 2.862959 | 823.812 |
| CN | CPA_H51 | CN | G47 | 3.500061 | 812.591 |
| AU | CPA_J62_63 | AU | G47 | 3.479847 | 811.907 |
| US | CPA_R93 | US | G47 | 2.858116 | 797.633 |
| CA | CPA_D35 | US | P3_S14 | 2.937849 | 784.508 |
| AU | CPA_H52 | AU | G47 | 3.465104 | 766.655 |
| ID | CPA_G46 | ID | G47 | 3.873976 | 707.577 |
| AU | CPA_N77 | AU | G47 | 3.445795 | 680.15 |
| CN | CPA_C13T15 | US | G47 | 2.867004 | 679.624 |
| US | CPA_S96 | US | G47 | 2.861026 | 677.897 |
| AU | CPA_I | AU | G47 | 3.521785 | 653.846 |
| CN | CPA_C29 | CN | G47 | 3.517818 | 647.32 |
| US | CPA_C28 | US | G47 | 2.869208 | 627.946 |
| CA | CPA_C20 | CA | F | 4.473996 | 608.494 |
| CN | CPA_N79 | CN | G47 | 3.510486 | 604.205 |
| GB | CPA_C20 | GB | M71 | 2.386655 | 538.989 |
| CN | CPA_K66 | CN | G47 | 3.504631 | 522.147 |
| US | CPA_S94 | US | G47 | 2.863248 | 481.254 |
| CA | CPA_D35 | CA | F | 16.07552 | 474.261 |
| AU | CPA_J61 | AU | G47 | 3.504231 | 439.464 |
| AU | CPA_S94 | AU | G47 | 3.484136 | 416.632 |
| RO | CPA_K64 | RO | G47 | 2.216315 | 408.782 |
| AU | CPA_K66 | AU | G47 | 3.490373 | 393.66 |
| US | CPA_R90T92 | US | G47 | 2.865032 | 390.435 |
| US | CPA_C21 | US | G47 | 2.867487 | 384.121 |
| BG | CPA_B | BG | C19 | 2.102353 | 373.797 |
| CN | CPA_B | CN | G47 | 3.515834 | 353.553 |
| RO | CPA_J62_63 | RO | G47 | 2.248163 | 334.974 |
| AU | CPA_H53 | AU | G47 | 3.411456 | 309.596 |
| CN | CPA_J58 | CN | G47 | 3.503108 | 304.863 |
| GB | CPA_C20 | GB | N80T82 | 2.395167 | 294 |
| CN | CPA_N77 | CN | G47 | 3.491151 | 289.051 |
| US | CPA_J59_60 | US | G47 | 2.870192 | 280.003 |
| GB | CPA_C20 | GB | P85 | 2.397075 | 270.634 |
| CN | CPA_C26 | US | G47 | 2.877619 | 265.851 |
| ID | CPA_D35 | ID | G46 | 2.763068 | 248.559 |
| ID | CPA_D35 | ID | S95 | 2.754456 | 242.755 |
| CN | CPA_E36 | CN | G47 | 3.517427 | 236.85 |
| GB | CPA_C20 | GB | G46 | 2.397299 | 236.195 |
| ID | CPA_B | ID | E37T39 | 9.783993 | 224.076 |
| CA | CPA_C20 | CA | C29 | 4.82576 | 217.473 |
| CA | CPA_C20 | CA | Q86 | 4.775235 | 211.13 |
| ID | CPA_D35 | ID | G47 | 2.597864 | 204.041 |
| ZA | CPA_K65 | ZA | G47 | 2.105284 | 193.907 |
| CA | CPA_C20 | CA | C19 | 4.874059 | 193.889 |
| AU | CPA_S96 | AU | G47 | 3.500952 | 193.76 |
| DK | CPA_J62_63 | DK | G47 | 23.90428 | 193.233 |
| GB | CPA_C20 | GB | O84 | 2.398038 | 188.769 |
| AU | CPA_J58 | AU | G47 | 3.505924 | 184.202 |
| CA | CPA_H51 | CA | C10T12 | 2.701106 | 183.721 |
| CA | CPA_C20 | CA | C16 | 4.739777 | 179.198 |
| ID | CPA_C10T12 | ID | G47 | 3.885546 | 176.667 |
| CA | CPA_M69_70 | US | G47 | 2.873537 | 169.539 |
| CN | CPA_J59_60 | CN | G47 | 3.511968 | 162.817 |
| MX | CPA_C25 | CA | F | 189.5417 | 160.397 |
| CA | CPA_C20 | CA | C25 | 4.914743 | 157.473 |
| ID | CPA_C13T15 | ID | G47 | 3.878186 | 154.738 |
| DK | CPA_N77 | DK | G47 | 23.59131 | 154.357 |
| CA | CPA_D35 | US | L | 2.929967 | 152.036 |
| ZA | CPA_E36 | ZA | G47 | 2.083216 | 147.955 |
| CA | CPA_C20 | CA | C31_32 | 4.782058 | 147.77 |
| US | CPA_C27 | US | G47 | 2.871907 | 146.734 |
| GB | CPA_C20 | GB | M72 | 2.393927 | 146.312 |
| CA | CPA_C29 | CA | F | 189.906 | 143.855 |
| DK | CPA_K64 | DK | G47 | 24.08178 | 135.849 |
| CN | CPA_C22 | US | G47 | 2.876131 | 135.414 |
| ID | CPA_C22 | ID | G47 | 3.877644 | 131.731 |
| CA | CPA_C20 | CA | S96 | 4.727427 | 121.58 |
| CA | CPA_C20 | CA | O84 | 4.791312 | 121.456 |
| CA | CPA_C22 | US | G47 | 2.888378 | 121.444 |
| CA | CPA_C20 | CA | N80T82 | 4.7523 | 112.532 |
| ID | CPA_H51 | ID | G47 | 3.875538 | 111.38 |
| AU | CPA_N79 | AU | G47 | 3.480681 | 111.096 |
| BR | CPA_C33 | BR | E37T39 | 23.81366 | 110.665 |
| CA | CPA_C20 | CA | C28 | 4.853866 | 106.673 |
| ID | CPA_S96 | ID | G47 | 3.856323 | 105.829 |
| RO | CPA_C27 | RO | H49 | 2.535123 | 100.891 |
| US | CPA_K64 | US | G47 | 2.838697 | 20228.8 |
| CN | CPA_L | CN | G47 | 3.481077 | 16180.52 |
| CN | CPA_K64 | CN | G47 | 3.481537 | 13815.85 |
| US | CPA_J62_63 | US | G47 | 2.830952 | 12618.31 |
| US | CPA_N77 | US | G47 | 2.81986 | 10782.07 |
| GB | CPA_C20 | GB | P3_S14 | 2.387603 | 9691.83 |
| CA | CPA_C25 | CA | F | 136.9456 | 7017.328 |
| US | CPA_P85 | US | G47 | 2.841851 | 6105.377 |
| CN | CPA_C13T15 | CN | G47 | 3.509119 | 5274.528 |
| US | CPA_I | US | G47 | 2.858456 | 5159.235 |
| US | CPA_C33 | US | G47 | 2.720443 | 5067.317 |
| US | CPA_C22 | US | G47 | 2.834539 | 4238.131 |
| RU | CPA_B | RU | P51G | 2.37694 | 4231.797 |
| US | CPA_C10T12 | US | G47 | 2.860481 | 4135.261 |
| US | CPA_F | US | G47 | 2.862721 | 4009.018 |
| CN | CPA_I | CN | G47 | 3.507936 | 3811.731 |
| IN | CPA_D35 | IN | O84 | 4.334481 | 3776.742 |
| ID | CPA_B | ID | D35 | 2.667001 | 3757.611 |
| US | CPA_C18 | US | G47 | 2.766087 | 3573.823 |
| CN | CPA_C18 | CN | G47 | 3.449796 | 3433.379 |
| IN | CPA_C19 | IN | O84 | 80.24901 | 3173.564 |
| US | CPA_K66 | US | G47 | 2.849134 | 3130.031 |
| CN | CPA_J61 | CN | G47 | 3.503288 | 2770.637 |
| US | CPA_C17 | US | G47 | 2.845952 | 2179.947 |
| CN | CPA_C17 | CN | G47 | 3.507281 | 2159.238 |
| AU | CPA_K64 | AU | G47 | 3.482323 | 1717.271 |
| US | CPA_C25 | CA | F | 189.2784 | 1596.602 |
| CN | CPA_H52 | CN | G47 | 3.499694 | 1594.24 |
| CA | CPA_C20 | CA | C22 | 4.471311 | 1467.041 |
| CN | CPA_S96 | CN | G47 | 3.504079 | 1260.462 |
| US | CPA_C16 | US | G47 | 2.847048 | 1236.476 |
| CN | CPA_H53 | CN | G47 | 3.443765 | 1070.715 |
| AU | CPA_F | AU | G47 | 3.497848 | 1016.938 |
| CN | CPA_C25 | CA | F | 190.7557 | 990.641 |
| RO | CPA_C29 | RO | H49 | 2.467052 | 947.444 |
| US | CPA_A03 | CA | C10T12 | 2.465997 | 922.696 |
| US | CPA_C26 | US | G47 | 2.868093 | 870.475 |
| US | CPA_C31_32 | US | G47 | 2.863049 | 855.433 |
| US | CPA_C25 | US | G47 | 2.852267 | 845.284 |
| US | CPA_K64 | CA | F | 188.3778 | 837.918 |
| US | CPA_J58 | US | G47 | 2.862959 | 823.812 |
| CN | CPA_H51 | CN | G47 | 3.500061 | 812.591 |
| AU | CPA_J62_63 | AU | G47 | 3.479847 | 811.907 |
| US | CPA_R93 | US | G47 | 2.858116 | 797.633 |
| CA | CPA_D35 | US | P3_S14 | 2.937849 | 784.508 |
| AU | CPA_H52 | AU | G47 | 3.465104 | 766.655 |
| ID | CPA_G46 | ID | G47 | 3.873976 | 707.577 |
| AU | CPA_N77 | AU | G47 | 3.445795 | 680.15 |
| CN | CPA_C13T15 | US | G47 | 2.867004 | 679.624 |
| US | CPA_S96 | US | G47 | 2.861026 | 677.897 |
| AU | CPA_I | AU | G47 | 3.521785 | 653.846 |
| CN | CPA_C29 | CN | G47 | 3.517818 | 647.32 |
| US | CPA_C28 | US | G47 | 2.869208 | 627.946 |
| CA | CPA_C20 | CA | F | 4.473996 | 608.494 |
| CN | CPA_N79 | CN | G47 | 3.510486 | 604.205 |
| GB | CPA_C20 | GB | M71 | 2.386655 | 538.989 |
| CN | CPA_K66 | CN | G47 | 3.504631 | 522.147 |
| US | CPA_S94 | US | G47 | 2.863248 | 481.254 |
| CA | CPA_D35 | CA | F | 16.07552 | 474.261 |
| AU | CPA_J61 | AU | G47 | 3.504231 | 439.464 |
| AU | CPA_S94 | AU | G47 | 3.484136 | 416.632 |
| RO | CPA_K64 | RO | G47 | 2.216315 | 408.782 |
| AU | CPA_K66 | AU | G47 | 3.490373 | 393.66 |
| US | CPA_R90T92 | US | G47 | 2.865032 | 390.435 |
| US | CPA_C21 | US | G47 | 2.867487 | 384.121 |
| BG | CPA_B | BG | C19 | 2.102353 | 373.797 |
| CN | CPA_B | CN | G47 | 3.515834 | 353.553 |
| RO | CPA_J62_63 | RO | G47 | 2.248163 | 334.974 |
| AU | CPA_H53 | AU | G47 | 3.411456 | 309.596 |
| CN | CPA_J58 | CN | G47 | 3.503108 | 304.863 |
| GB | CPA_C20 | GB | N80T82 | 2.395167 | 294 |
| CN | CPA_N77 | CN | G47 | 3.491151 | 289.051 |
| US | CPA_J59_60 | US | G47 | 2.870192 | 280.003 |
| GB | CPA_C20 | GB | P85 | 2.397075 | 270.634 |
| CN | CPA_C26 | US | G47 | 2.877619 | 265.851 |
| ID | CPA_D35 | ID | G46 | 2.763068 | 248.559 |
| ID | CPA_D35 | ID | S95 | 2.754456 | 242.755 |
| CN | CPA_E36 | CN | G47 | 3.517427 | 236.85 |
| GB | CPA_C20 | GB | G46 | 2.397299 | 236.195 |
| ID | CPA_B | ID | E37T39 | 9.783993 | 224.076 |
| CA | CPA_C20 | CA | C29 | 4.82576 | 217.473 |
| CA | CPA_C20 | CA | Q86 | 4.775235 | 211.13 |
| ID | CPA_D35 | ID | G47 | 2.597864 | 204.041 |
| ZA | CPA_K65 | ZA | G47 | 2.105284 | 193.907 |
| CA | CPA_C20 | CA | C19 | 4.874059 | 193.889 |
| AU | CPA_S96 | AU | G47 | 3.500952 | 193.76 |
| DK | CPA_J62_63 | DK | G47 | 23.90428 | 193.233 |
| GB | CPA_C20 | GB | O84 | 2.398038 | 188.769 |
| AU | CPA_J58 | AU | G47 | 3.505924 | 184.202 |
| CA | CPA_H51 | CA | C10T12 | 2.701106 | 183.721 |
| CA | CPA_C20 | CA | C16 | 4.739777 | 179.198 |
| ID | CPA_C10T12 | ID | G47 | 3.885546 | 176.667 |
| CA | CPA_M69_70 | US | G47 | 2.873537 | 169.539 |
| CN | CPA_J59_60 | CN | G47 | 3.511968 | 162.817 |
| MX | CPA_C25 | CA | F | 189.5417 | 160.397 |
| CA | CPA_C20 | CA | C25 | 4.914743 | 157.473 |
| ID | CPA_C13T15 | ID | G47 | 3.878186 | 154.738 |
| DK | CPA_N77 | DK | G47 | 23.59131 | 154.357 |
| CA | CPA_D35 | US | L | 2.929967 | 152.036 |
| ZA | CPA_E36 | ZA | G47 | 2.083216 | 147.955 |
| CA | CPA_C20 | CA | C31_32 | 4.782058 | 147.77 |
| US | CPA_C27 | US | G47 | 2.871907 | 146.734 |
| GB | CPA_C20 | GB | M72 | 2.393927 | 146.312 |
| CA | CPA_C29 | CA | F | 189.906 | 143.855 |
| DK | CPA_K64 | DK | G47 | 24.08178 | 135.849 |
| CN | CPA_C22 | US | G47 | 2.876131 | 135.414 |
| ID | CPA_C22 | ID | G47 | 3.877644 | 131.731 |
| CA | CPA_C20 | CA | S96 | 4.727427 | 121.58 |
| CA | CPA_C20 | CA | O84 | 4.791312 | 121.456 |
| CA | CPA_C22 | US | G47 | 2.888378 | 121.444 |
| CA | CPA_C20 | CA | N80T82 | 4.7523 | 112.532 |
| ID | CPA_H51 | ID | G47 | 3.875538 | 111.38 |
| AU | CPA_N79 | AU | G47 | 3.480681 | 111.096 |
| BR | CPA_C33 | BR | E37T39 | 23.81366 | 110.665 |
| CA | CPA_C20 | CA | C28 | 4.853866 | 106.673 |
| ID | CPA_S96 | ID | G47 | 3.856323 | 105.829 |
| RO | CPA_C27 | RO | H49 | 2.535123 | 100.891 |
| … | … | … | … | … | … |
| … | … | … | … | … | … |
| MT | CPA_C19 | RW | P3_S13 | 2.135604 | 0 |
| CY | CPA_B | SI | P5M | 2.875691 | -0.002 |
| CY | CPA_B | HU | P5M | 2.883374 | -0.028 |
| CY | CPA_B | GR | P5M | 2.878904 | -0.034 |
| CY | CPA_B | SE | P5M | 2.892981 | -0.045 |
| CY | CPA_B | MT | P5M | 2.840287 | -0.047 |
| CH | CPA_B | DK | P5M | 3.181018 | -0.071 |
| CY | CPA_C17 | GR | P5M | 2.431223 | -0.093 |
| CH | CPA_B | CH | P5M | 3.177003 | -10.35 |

There are 91 large ratios above 352, as well as 45 ratios between 200-352, which probably reflects some small degree of freedom to match some of the searched values. All original values are below 1.3 and most of them are actually marginal values. Another 196 are between 100-200, and all other ratios are below 100, being on the whole less than 1755 (of the total 4,107,904 non-zero values) the ratios above ten. Another way that we could look at this is that there are also only nine ratios below 0.19 (and of this, two are negative, relative to P5M, the changes in inventories and acquisition less disposals of valuables), for original values all below $\left| 0.03 \right|$. Additionally, there are 53 additional ratios between 0.19 and 0.2 for LV_CPA_C19 (with maximum original value 0.187) and other 15 ratios between 0.2 and 0.28. All in all, there are ~1990 ratios below 0.5, with average absolute value of 4.702.

As all checks above performed for the Use table (the Supply has zero deviations), we compute the Weighted Absolute Percentage Error (WAPE, defined in equation 7) representing the discrepancies between all the Agg_FIG-E and the original Agg_FIG, for which we obtain **0.48%** (after the final iteration), converging as shown in Figure 2. This indeed shows that the two tables are much alike.

${WAPE\_u}^{FIG-e,\mathrm{FIG}}=\sum_{k=1}^{m} \sum_{l=1}^{n} \left( \frac{\left| \tilde{u}_{kl}^{FIG-e}-u_{kl}^{\mathrm{FIG}} \right|}{\sum_{k=1}^{m} \sum_{l=1}^{n} u_{kl}^{\mathrm{FIG}}} \right)\times100$ (3)

In the WAPE, $\tilde{u}_{kl}^{FIG-e}$ are the values in FIGARO-E3 and $u_{kl}^{\mathrm{FIG}}$ the values in FIGARO, absolute differences which are put in relative terms with respect to the sum of all values.

Figure 2: WAPE of the Use table after each adjustment (of application of the ratio + GRAS procedure)

Finally, we move into the comparison (of deviations) of the MR Symmetric IO tables, not only of FIGARO-E3 with FIGARO but also of other MRIO databases with respect to FIGARO data.

Following the same equation 7 of WAPE above, but for the symmetric tables’ values (built with Model D, instead of looking at the Use table as above), for FIGARO-E3 for the intermediate use part we obtain a WAPE of 0.7%, for the Final Demand more than 0.2%, for the value added 0.1%, totalling 1%.

As shown in Table 4, the differences between the OECD Inter-Country Input-Output tables (OECD, 2023), GLORIA (Lenzen et al., 2022) and EXIOBASE (Stadler et al., 2018a), on the one hand, and FIGARO, on the other, are much higher than those between FIGARO-E3 and FIGARO. This shows the extent of deviations from ‘official statistics’ for the year 2015 when these are interpreted as being represented in FIGARO.

Table 4: WAPE results for different MRIO databases

| **Database** | **Intermediate use** | **Value Added** | **Final Demand** | **Total** |
| --- | --- | --- | --- | --- |
| FIGARO-E3 | 1% | 0% | 0% | 1% |
| ICIO | 16% | 6% | 12% | 34% |
| GLORIA | 16% | 12% | 11% | 39% |
| EXIOBASE | 33% | 17% | 33% | 83% |

As shown in Figure 3, the ‘rest-of-world’ region, the US, China and Japan are the countries in which the highest differences arise.

Figure 3: WAPE results per country


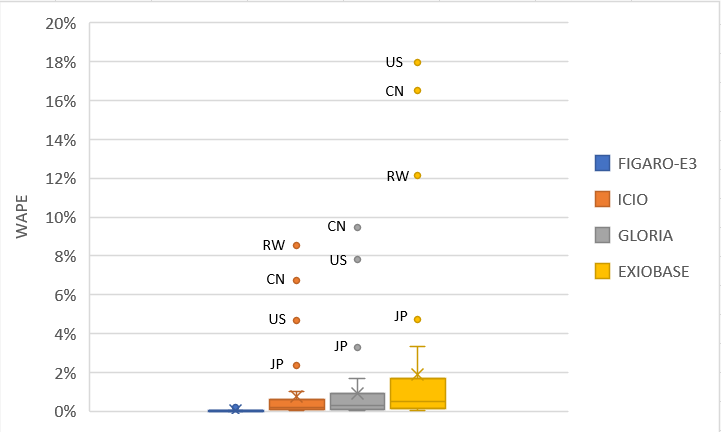


The detailed comparison by countries for FIGARO-E3 is shown in the following table, where the largest WAPE differences are found for China and USA, followed at a distance of the Rest of the World (FIGW1) and India. Relative to the total output (column of WAPE WAPE/Output in trillion €), we may see that the largest WAPEs are found for Croatia, Bulgaria, Ireland, Slovakia, Lithuania, Malta and Slovenia.

Table 5: WAPE results for the different countries of the Intermediate use and final demands of the Symmetric (Model D type) FIGARO-E3 wrt FIGARO IO table

| **Country** | **WAPE** | **WAPE/Output (trillion €)** | **Country** | **WAPE** | **WAPE/Output (trillion €)** | **Country** | **WAPE** | **WAPE/Output (trillion €)** |
| --- | --- | --- | --- | --- | --- | --- | --- | --- |
| AU | 0.029564 | 0.087169 | GB | 0.022105 | 0.030544 | NO | 0.003801 | 0.041733 |
| AT | 0.00413 | 0.042702 | GR | 0.003748 | 0.080456 | PL | 0.009826 | 0.077062 |
| BE | 0.009128 | 0.072561 | HR | 0.001621 | 0.134478 | PT | 0.00286 | 0.057647 |
| BG | 0.001627 | 0.114667 | HU | 0.002633 | 0.080426 | RO | 0.003874 | 0.081627 |
| BR | 0.028876 | 0.065199 | ID | 0.010546 | 0.046981 | RU | 0.023854 | 0.06803 |
| CA | 0.033927 | 0.086869 | IN | 0.043843 | 0.076886 | SK | 0.002701 | 0.105368 |
| CH | 0.005593 | 0.030687 | IE | 0.007855 | 0.111227 | SI | 0.000981 | 0.090158 |
| CN | 0.246111 | 0.06485 | IT | 0.028425 | 0.059797 | SE | 0.004142 | 0.032968 |
| CY | 0.000345 | 0.069161 | JP | 0.036776 | 0.031718 | TR | 0.012932 | 0.058144 |
| CZ | 0.003947 | 0.071846 | KR | 0.022465 | 0.052902 | US | 0.149767 | 0.033054 |
| DE | 0.031003 | 0.037035 | LT | 0.001012 | 0.09868 | ZA | 0.006619 | 0.077979 |
| DK | 0.00504 | 0.069001 | LU | 0.000763 | 0.035554 | AR | 0.007378 | 0.048743 |
| ES | 0.012773 | 0.043024 | LV | 0.000561 | 0.077777 | FIGW1 | 0.062254 | 0.025896 |
| EE | 0.00041 | 0.06756 | MX | 0.018282 | 0.064511 | SA | 0.004305 | 0.028941 |
| FI | 0.001942 | 0.032427 | MT | 0.000347 | 0.097912 | **TIU+FD*** | **0.936546** | **0.046921** |
| FR | 0.014802 | 0.024413 | NL | 0.011051 | 0.055894 |  |  |  |

* TIU+FD: Total Intermediate Use + Final Demand

And analogously, we may compare by industries for FIGARO-E3, as shown in the following table, where the highest WAPEs are driven, apart from the large P3_S14 (household consumption) category, by G47, C19 and C20 industries. In relative terms to the total output, the differences of C19 are also the largest, followed by E37T39, C20, C22 and G47. In the final demand components in relative terms to the total of the category, the differences in P5M stand out.

Table 6: WAPE results for the different industries of the Intermediate use part of the Symmetric (Model D type) FIGARO-E3 wrt FIGARO IO table

| **Industry** | **WAPE** | **WAPE/Output (trillion €)** | **Industry** | **WAPE** | **WAPE/Output (trillion €)** |
| --- | --- | --- | --- | --- | --- |
| A01 | 0.023 | 0.005962 | H51 | 0.002 | 0.003629 |
| A02 | 0.001 | 0.002092 | H52 | 0.004 | 0.002679 |
| A03 | 0.001 | 0.00469 | H53 | 0.001 | 0.001851 |
| B | 0.014 | 0.005285 | I | 0.016 | 0.00502 |
| C10T12 | 0.033 | 0.006141 | J58 | 0.001 | 0.002111 |
| C13T15 | 0.013 | 0.00646 | J59_60 | 0.001 | 0.001348 |
| C16 | 0.003 | 0.003943 | J61 | 0.003 | 0.00149 |
| C17 | 0.007 | 0.008909 | J62_63 | 0.003 | 0.00125 |
| C18 | 0.004 | 0.010669 | K64 | 0.004 | 0.000974 |
| C19 | 0.077 | 0.032322 | K65 | 0.002 | 0.001372 |
| C20 | 0.071 | 0.022806 | K66 | 0.001 | 0.00097 |
| C21 | 0.009 | 0.007806 | L | 0.010 | 0.001226 |
| C22 | 0.036 | 0.022634 | M69_70 | 0.004 | 0.001367 |
| C23 | 0.009 | 0.00645 | M71 | 0.003 | 0.002388 |
| C24 | 0.035 | 0.010206 | M72 | 0.006 | 0.007177 |
| C25 | 0.011 | 0.005572 | M73 | 0.001 | 0.002027 |
| C26 | 0.012 | 0.004168 | M74_75 | 0.003 | 0.004476 |
| C27 | 0.007 | 0.004138 | N77 | 0.002 | 0.001899 |
| C28 | 0.011 | 0.003763 | N78 | 0.001 | 0.00104 |
| C29 | 0.011 | 0.003133 | N79 | 0.001 | 0.002007 |
| C30 | 0.003 | 0.003045 | N80T82 | 0.010 | 0.005626 |
| C31_32 | 0.006 | 0.005143 | O84 | 0.014 | 0.002027 |
| C33 | 0.001 | 0.002695 | P85 | 0.009 | 0.001927 |
| D35 | 0.028 | 0.007867 | Q86 | 0.009 | 0.001842 |
| E36 | 0.002 | 0.010914 | Q87_88 | 0.002 | 0.001376 |
| E37T39 | 0.016 | 0.023873 | R90T92 | 0.001 | 0.001623 |
| F | 0.039 | 0.004402 | R93 | 0.001 | 0.002324 |
| G45 | 0.005 | 0.003382 | S94 | 0.001 | 0.002125 |
| G46 | 0.016 | 0.002539 | S95 | 0.001 | 0.004212 |
| G47 | 0.082 | 0.018561 | S96 | 0.002 | 0.002904 |
| H49 | 0.008 | 0.002723 | T | 0.000 | 1.26E-06 |
| H50 | 0.005 | 0.007191 | **TIU** | **0.718** | **0.005445** |

Table 7: WAPE results for the final demand part of the Symmetric (Model D type) FIGARO-E3 wrt FIGARO IO table

| **Category** | **WAPE** | **WAPE/Category total (trillion €)** |
| --- | --- | --- |
| P3_S14 | 0.165 | 0.004364 |
| P3_S15 | 0.000 | 0.000406 |
| P3_S13 | 0.010 | 0.000901 |
| P51G | 0.031 | 0.001815 |
| P5M | 0.011 | 0.023562 |
| **FD** | **0.218** | **0.003224** |

### Labour data

- 1. **Methodology**

**Benchmark of OECD data complemented with EXIOBASE data**

The main employment data benchmark is the OECD data, in particular the “TiVA Indicators: 2021 edition,” specifically the variable "EMPN" representing total employment (jobs/persons)^^[[1]](#footnote-1)^^ for OECD countries. This data provides information for 54 out of 70 OECD countries, but more importantly, for all those 45 countries in FIGARO, but no information for “other countries” or TOTAL to complete the “Rest of the World”.

Hence the ROW region requires a different choice. In order to avoid deviating from the EXIOBASE values without enough justified arguments, the ROW of FIGARO-E3 is constructed as five WW (the five rest of the world regions of EXIO) +TWN-SAU-ARG (Taiwan, Saudi Arabia, and Argentina).

The OECD data exists for $l=1\ldots36$ industries (plus some additional aggregates, which we basically used for verification or checks) and it is consistent with Eurostat data at 21 NACE categories (differences around 5%)^^[[2]](#footnote-2)^^.

The EXIOBASE employment data (Stadler et al., 2018b)^^[[3]](#footnote-3)^^ is derived from Eurostat, ILO, and OECD statistics and offers a robust basis for disaggregated departure point, though the differences between EXIOBASE and OECD are generally around 6%, with EXIOBASE often slightly lower than OECD figures. The concept indicated in EXIOBASE is Number of persons engaged (thousands)^^[[4]](#footnote-4)^^.

Accordingly, our initial split (into the 6 skill-gender combinations) of the OECD data is performed directly from the 2015 EXIOBASE sub-activities.

**Disaggregation to FIGARO-E3**

The OECD data was directly split into the FIGARO-E3 framework using the 2015 EXIOBASE industries. The data from OECD for $l=1\ldots36$ industries is the benchmark, and the EXIOBASE more disaggregated industries essentially lead to FIGARO-E3 detail. The same principles than for disaggregating the Supply and Use tables prevail here. In the event that the (FIGARO) OECD category was more disaggregated than the original EXIOBASE, the OECD value prevails. Since then the FIGARO-E3 is more detailed in a few cases than EXIOBASE^^[[5]](#footnote-5)^^, here the EXIOBASE structures need to be disaggregated to reach FIGARO-e classification (for the labour accounts, this basically implies assuming the same coefficients per unit of output for the subindustries).

**Split by Gender and Skill**

Following with the use of EXIOBASE for the split, this is also crucial for the distinction of employment by gender and by skill type. The analogous data, the detail of shares, of the OECD data provided in the “TiVA indicators: 2021 edition”, is the “Trade in Employment by Characteristics”, available for 43 countries and $l=1\ldots11$ industries. Apart from splitting by male/female, this data incorporates for most countries two types of split by High, Medium and Low (H-M-L) skills; one based on occupation and another one on education attained. In the finally provided version, we obtain two types of results: one matching the total EMPN and the H-M-L by occupation (OCC) ICIO data; and the one matching the total EMPN and the H-M-L by education attained (EDU) ICIO data. Since the ICIO data does not provide the mix of gender-skill combination, for these two last types of results we depart from the EXIOBASE gender-skill combined structure. The FIGARO-E3 industrial level keeps the FIGARO detail (so all OECD detail is preserved) plus it incorporates the EXIOBASE detail. We impose the OECD data constraints both at 11 industries (gender and skill level) and 36 industries (sectoral level $l$), applying, as described in the final subsection, a RAS balancing method to match those totals. In practice though, given that the sectoral constraint is given at 36 industries, we first develop the skill and gender splits at 36 industries, which will be our benchmarks and constraints.

We can also briefly comment on the case mix in terms of data availability. The countries for which EMPN (total) data is available, but not the gender and skill detail are Argentina (ARG), Brazil (BRA), China (CHN), India (IND), Indonesia (IDN), Russia (RUS), Saudi Arabia (SAU) and South Africa (ZAF). For all countries we have the total EMPN value from the OECD (TiVA) statistics and minimal additional info was obtained from additional sources (EUKLEMS^^[[6]](#footnote-6)^^/WorldKLEMS, Ark & Jäger, 2017; Jäger, 2017), especially those used to build EXIOBASE (Eurostat, ILO^^[[7]](#footnote-7)^^ and OECD’s Statistics^^[[8]](#footnote-8)^^). So EXIOBASE only gender and skill structures are needed: we use country specific structures for BRA, CHN, IND, IDN, RUS, ZAF (plus KOR and MEX lacking OCC info and JPN lacking EDU info), and general regions structures (WWL and WWM respectively) for ARG and SAU.

**Benchmarking**

We split the OECD (ICIO) data based on the EXIOBASE data for all countries. For the cases when there was a one-to-one correspondence (both in industries and regions) between OECD and FIGARO-E3 (which is a common constructed classification from FIGARO and EXIOBASE), the benchmark is straightforward:

$\tilde{e}_{i}^{u, FIG-e}=e_{l}^{s OECD}$ (4)

Where $i=1\ldots176$industries; $u=1\ldots46$ regions.

With $l=1\ldots36$ industries, $s=1\ldots70$ (52 with employment data for 2015) regions.

For the cases when there is not a one-to-one correspondence in regions, between OECD and FIGARO-E3, the values are introduced firstly as all others of the OECD, so that we have $e_{l}^{u OECD}$. In other words, the $s$ are firstly matched to $u=1\ldots46$ regions, so that we can write the equations just with $u$ and the only matching remaining is that of industries. This implies that when the FIGARO-E3 data is more disaggregated than in the original EXIOBASE (EXIOBASE accounts: A_FURN, A_TDTR, A_TAUX, A_PTEL, A_OBUS, A_HEAL, A_RECR) the OECD value prevails if available.

For most other industries that do not have a one-to-one correspondence, the OECD data is less detailed than the EXIOBASE data and, hence, than the finally desired FIGARO-E3 data^^[[9]](#footnote-9)^^. As for the case of the supply and use input-output tables we may call $p$ the number of FIGARO industries which correspond to a certain FIGARO industry. For example, there are 17 FIGARO-E3 industries which serve to disaggregate the agrarian OECD industry “D01T03 Agriculture”, or conversely seen, 17 FIGARO-E3 industries which are informed from one OECD industry (and 17 EXIOBASE detailed industries).

So, for the cases when $n$ industries in FIGARO-E3 (whose initial points are obtained from EXIOBASE) correspond to one industry in OECD, the components of the OECD employment dataset are disaggregated using the structures of the EXIOBASE, which are converted to the common classification of $e_{i}^{u FIG-e}$.

$\tilde{e}_{i}^{u FIG-e}=e_{l}^{u OECD}\left( e_{i}^{u FIG-e}/\sum_{i=l_{1}}^{l_{p}} e_{i}^{u FIG-e} \right)$ (5)

Where $e_{l}^{u OECD}$ represents the OECD original data by country $u$ and industry $l$ and $\tilde{e}_{i}^{u FIG-e}$ the FIGARO-E3 by country $u$ and industry $i$.

Let’s denote these characteristics, skill $w$ and gender $x.$ From EXIOBASE there is $e_{i,x}^{u, w EXIO}$, while for the OECD data we have $e_{l}^{u, w OECD}$ with detail by skills and $e_{l,x}^{u OECD}$ with detail by gender, but there is not detail by skill and gender ($e_{i,x}^{u, w O}$).

To adjust by these characteristics, we depart from the original split by skill and gender from EXIOBASE, making first the very direct correspondence between EXIOBASE and FIGARO-E3 industries.

$\tilde{e}_{i,x}^{u,w FIG-e}=e_{i,x}^{u,w EXIO}\left( \tilde{e}_{i}^{u FIG-e}/\sum_{w} \sum_{x} e_{i,x}^{u,w EXIO} \right)$ (6)

**Bi-Proportional Adjustment (RAS) Procedure**

Figure 4: Schematic representation of the 6 skill-gender combinations and aggregations/constraints


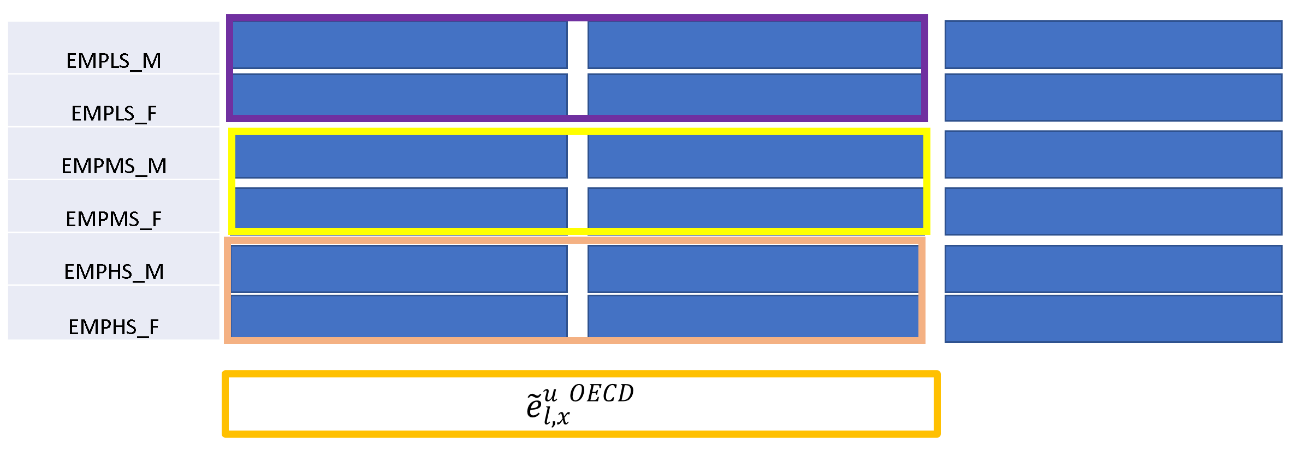

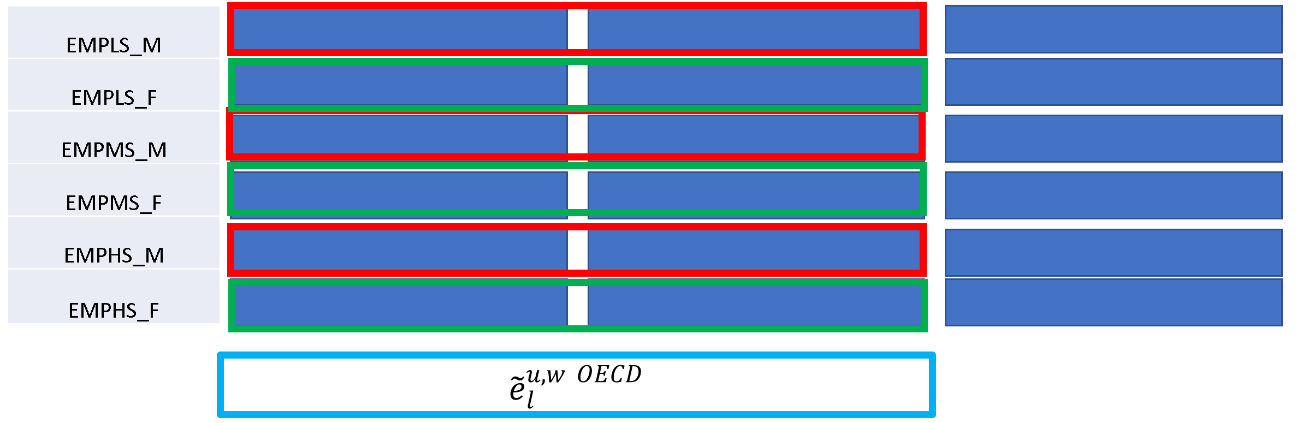


The RAS type procedure is implemented with the constraints below for each $l=1\ldots36$ industry. As shown in Figure 4, the aggregates that we obtain in the initial distribution can be represented both as skill aggregates of three pairs of rows (purple, yellow and orange), or as gender aggregates of 2 trios of rows (red and green). Given the benchmarks, the constraints imposed, the goal is to comply with both equation 11 and 12.

$\sum_{i=l_{1}}^{l_{p}} \sum_{w} \tilde{e}_{i,x}^{u,w FIG-e}= \tilde{e}_{l,x}^{u OECD}$ (7)

$\sum_{i=l_{1}}^{l_{p}} \sum_{x} \tilde{e}_{i,x}^{u,w FIG-e}= \tilde{e}_{l}^{u, w OECD}$ (8)

The iterative procedure solves for the skill and gender constraints (13 and 14), which can be thought of or interpreted as row and column constraints if one would reorganize the cells, the skill-gender combinations, for each $l$.

Figure 5: Schematic representation of how the main constraints may be reorganized/interpreted as row and column constraints


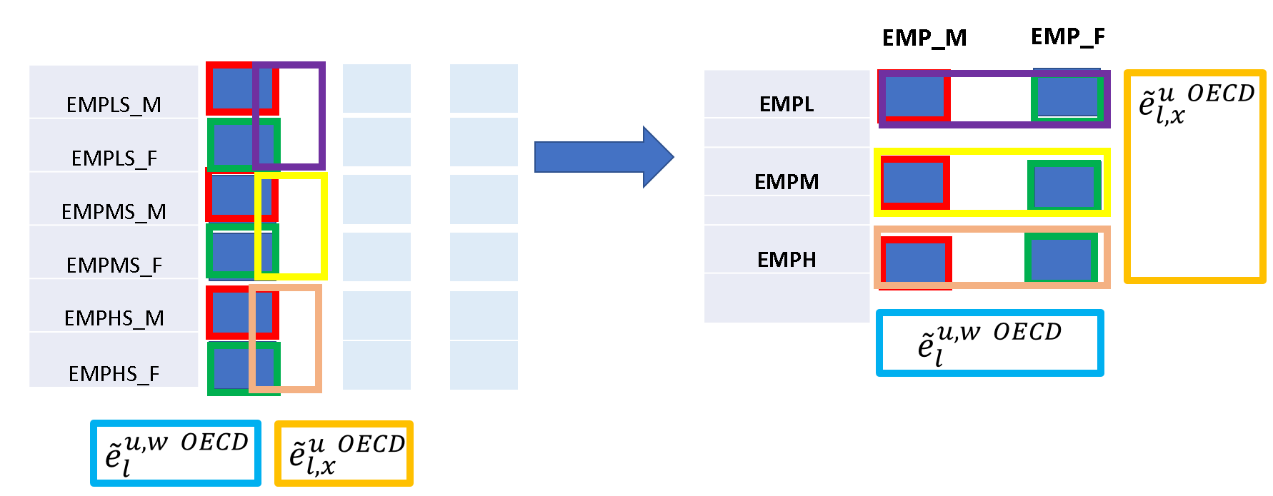


$\tilde{e}_{i,x}^{u,w FIG-e}=\tilde{e}_{l,x}^{u OECD}\left( \tilde{e}_{i,x}^{u,w FIG-e}/\sum_{i=l_{1}}^{l_{p}} \sum_{w} \tilde{e}_{i,x}^{u,w FIG-e} \right)$ (9)

$\tilde{e}_{i,x}^{u,w FIG-e}=\tilde{e}_{l}^{u,w OECD}\left( \tilde{e}_{i,x}^{u,w FIG-e}/\sum_{i=l_{1}}^{l_{p}} \sum_{x} \tilde{e}_{i,x}^{u,w FIG-e} \right)$ (10)

Avoidance of outliers in wage (compensation of employees to employment) and employment intensity (employment to output) ratios

At this point we only have one constraint left to explain, which is the avoidance of too large or too small wage and employment intensity ratios. These are based, fixed at sectoral level at 50 times (above, and below) the global ratios.

2 versions for the skill type split

We could have explained all the process not just for $OECD$ data but for something like $OECD\_OCC$ and $OECD\_EDU$, indicating that the split by skill relies on two different sets of information provided by the OECD for the skill split, one based on occupations, and one based on education attainment. In other words, indeed there is not only a unique form of OECD data for skill $e_{l}^{u, w OECD}$. There is actually a$w_{1}$ based on occupations, and a $w_{2}$ based on education (the two as alternatives from the OECD data).

Indeed, after the main iterative balancing of equations 13 and 14 (respectively for w1 and 21) both versions ($OECD\_OCC$ and $OECD\_EDU$) comply with the totals for every industry $l$, by gender and by skill (respectively their own constraints). But the totals (aggregated skill and gender) for each FIGARO E3 industry in general differ: $\tilde{e}_{i}^{u FIG-e}=\sum_{w1} \sum_{x} \tilde{e}_{i,x}^{u,w1 FIG-e}\neq\sum_{w2} \sum_{x} \tilde{e}_{i,x}^{u,w2 FIG-e}$

The reason is that there is no initial constraint on the total labour by subindustry $i$. At this point we want to have common aggregates of the two versions, so we impose for all $i$ the average sum by gender reached in the “quasi final” (not improving the convergence) iteration for gender ($\sum_{x} \tilde{e}_{i,x}^{u,w1 FIG-e}+\sum_{x} \tilde{e}_{i,x}^{u,w2 FIG-e})/2$.

The final balancing has now this new gender constraint at the FIGARO E3 industry $i$ detail and the own skill constraint for each (OCC and EDU) case, complying strictly with the gender constraint.

All in all, we get two types of results^^[[10]](#footnote-10)^^: making use of the OECD skills and gender OECD totals, one based on the skill by education (EDU), and another by occupation (OCC).

### Illustrative comparisons of the OECD, Eurostat and EXIOBASE data

Figure 6 illustrates the OECD and Eurostat (Total) Employment data comparison.

Figure 6: OECD and Eurostat (Total) Employment data comparison.

Note: Eurostat data is smaller notably for Switzerland (CH), Luxembourg (LU), Bulgaria (BG) and Greece (EL), existing higher than 10% differences for all of them.

Source: own calculation

Figure 7 illustrates the OECD and EXIOBASE (Total) Employment data comparison. It stands out the large positive difference (i.e., much higher value in EXIOBASE than in the OECD data) of Mexico (MX), existing also some large negative differences for Luxembourg (LU, as also between OECD and Eurostat data), Japan (JP), Russia (RU), South Africa (ZA), Brazil (BR), India (IN) or the Netherlands (NL), showing all absolute (negative %, i.e., significantly smaller values in EXIOBASE) percentage differences above 15%.

Figure 7: OECD and EXIOBASE (Total) Employment data comparison

Source: own calculation

### Energy and air emissions

FIGARO-E3 contains two main energy extensions: primary energy supply and net energy use. Nonetheless, these require data on gross energy supply and gross energy use as precursors. Likewise, the GHG accounts require data on emission-relevant energy use. Primary energy supply is a subset of gross energy supply, while net energy use and emission relevant energy use are subsets of gross energy use. The sum of final energy use, energy industry own use and energy losses equals net energy use. Non-energy use is a subset of final energy use. The definition of each indicator is shown in Table 8. Additional information can be found in (Usubiaga-Liaño et al., 2021).

Table 8: Selected energy indicators to produce energy and GHG emission accounts

| **Side** | **Energy indicator** | **Definition** |
| --- | --- | --- |
| Supply | Gross energy supply (GES) | Supply of all energy products, primary or secondary, by domestic industries. It does not include imports in MRIO format. |
|  | Primary energy supply (PES) | Supply of energy products extracted from the environment by domestic industries. It does not include imports in MRIO format. |
| Use | Gross energy use (GEU) | Use of all energy products, primary or secondary by domestic industries and final consumers such as households. It does not include exports in MRIO format. |
|  | Net energy use (NEU) | Use of energy products by domestic end users (*), including the losses incurred during transformation, distribution, transmission and transport, but excluding exports. |
|  | Final energy use (FEU) | Use of energy products by non-energy end users. |
|  | Energy industry own use (EOU) | Final energy consumption by the energy industry. |
|  | Energy losses (LOSS) | Transformation, distribution, transmission and transport losses. It includes stock changes and statistical differences. |
|  | Non-energy use (NENE) | Energy use for non-energetic purposes. |
|  | Emission-relevant energy use (EREU) | Use of all energy products that lead to air emissions by domestic industries and final consumers such as households. |

(*): End users represent the activities by industries and households where energy is used but not transformed into other secondary energy products.

Source: Based on (Usubiaga-Liaño et al., 2021)

The general approach used in shown in Figure 8.

Figure 8: General approach used to produce the energy extensions

Acronyms: c=countries; EP: energy product; EF: energy flow; PES: primary energy supply; GES: gross energy supply; GEU: gross energy use; EREU; emission-relevant energy use; NEU: net energy use; NI: natural inputs; I: industries

As shown in the left-hand side, the IEA energy balances of the year 2015 (IEA, 2017a, 2017b) are processed and some minor modifications are undertaken (e.g. correct inputs in some transformation processes where efficiencies are higher than 100%, to balance trade data at the global level or to gap-fill the energy use data in some industries when breaks in time series exist). After that, the balances are arranged in gross energy supply and use tables. All in all, we produced gross energy supply and use tables for 146 countries (later on aggregated to 45 countries plus a rest-of-world region), each of which represents 63 energy products and 85 energy flows as defined in the original IEA documentation (IEA, 2017c). The country classification is shown in the supplementary material.

The main two steps involved in generating energy accounts require aligning the IEA energy balances with the residence principle (Usubiaga & Acosta-Fernández, 2015), and allocating the IEA energy flows and products to the FIGARO-E3 industry (176), final consumer (5) and product (213) classifications.

As mentioned above, the first step after splitting the IEA data into energy supply and use tables is to bridge the gap between the territory and the residence principles. As explained in (Usubiaga & Acosta-Fernández, 2015) and in the supplementary material of (Stadler et al., 2018b), the IEA data is compiled according to the territory principle and therefore considers the activities taking place within national territory and offshore areas over which the country has jurisdiction. In contrast, the energy extensions should be compiled according to the residence principle, which does not consider where activities take place, but rather the residence of the agent undertaking those activities. In practice, there is an overlap between most of the activities accounted for given that these are undertaken by resident agents within their territory. Nonetheless, there are some aspects related to transport activities that need to be captured properly to make the bridge. These refer to international aviation, navigation and road transport. Figure 9 shows the differences between both principles in practical terms.

Figure 9: Differences between the territory and residence principles applied to air emissions


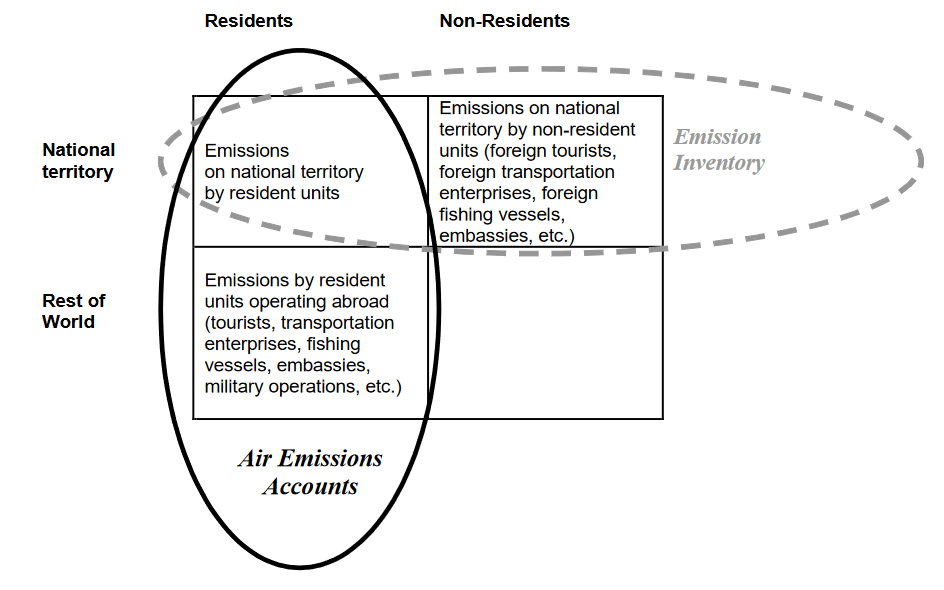


Source: (Usubiaga & Acosta-Fernández, 2015)

The approach used to bridge this gap was originally described in (Stadler et al., 2018b). It required to add the energy use related to marine and aviation bunkers to the energy supply and use tables, as well as to correct the road transportation data to consider the ‘fuel tourism effect’ in households and freight transport (Banfi et al., 2005; Jansen & Jonker, 2018), which are particularly relevant in Europe due to cross-country differences in fuel taxation (Wlazlowski et al., 2009). For international aviation, we use estimates of the territory-residence bridging items for 186 countries compiled by the OECD (Clarke et al., 2022). In absence of similar data for international navigation, we use a relatively simple model documented in (Stadler et al., 2018b). Lastly, in the case of road transport, it has been assumed that in non-European countries the differences between road transport taking place within a territory and road transport undertaken by resident agents is similar, and therefore the net effect is negligible. This is known not to hold in European countries because of their cohesiveness and different fuel prices (Wlazlowski et al., 2009). As a result, there are countries like Luxembourg in which the fuel sold within the territory is mostly consumed by non-residents outside of the country. To deal with this effect, bridging items reported by European countries (Eurostat, 2022b) has been used. To fill data gaps, the net effect of the territory-residence principle for road transport has been assumed to be zero in Europe as a whole. In other words, while the effect in countries might be possible or negative, as a whole, Europe is considered a closed system.

The data described in the previous paragraph is integrated in the IEA supply and use tables to transform them to the residence principle. The resulting tables are structured around 63 energy products and 89 energy flows (see supplementary material). From these tables, net energy use and emission-relevant use data is produced by selecting the specific energy products and energy flows that are relevant to each of them following the definitions in Table 8.

Finally, the resulting data is allocated from IEA energy products and flows to FIGARO-E3 products, industries and final consumption categories to create the energy extensions. In most cases, the allocation is done from one energy flow to one or several industries. In the latter case, the allocation is done based on monetary proportions as given in the monetary supply and use tables. There are a few flows that are allocated differently. These refer to electricity supply and use, which is allocated depending on the technology used (coal, gas, solar, wind, etc.), or road transport, which needs to be split between the road transport service industries (e.g. public and private passenger transport activities, freight transport services), private household transport, and road transport undertaken directly by industries (e.g. with vehicles they own). To that end, we have used the consumption of relevant fuels in the intermediate and final demand matrices to split the energy use related to road transport. The complete allocation approach, including the type of allocation used for each flow is described in (Stadler et al., 2018b). The specifics for net energy use are described in (Usubiaga-Liaño et al., 2020). Final energy use, energy industry own use and energy losses are subcomponents of net energy use. Finally, the primary energy supply extension, which represents 19 types of natural inputs, is generated by selecting the primary energy products from the gross energy supply dataset. While, in most cases the allocation is done directly, the extraction of the raw materials used for nuclear fuel is allocated to the country of origin using auxiliary data. The correspondence tables between energy flows and the FIGARO-E3 classification are shown in the supplementary material.

The GHG emission accounts cover the following pollutants: CO_2_, CH_4_, N_2_O and F-gases, which represent a compendium of the following substances (CF_4_, C_2_F_6_, C_3_F_8_, c-C_4_F_8_, C_4_F_10_, C_5_F_12_, C_6_F_14_, HCFC-141b, HCFC-142b, HFC-125, HFC-134, HFC-134a, HFC-143, HFC-143a, HFC-152a, HFC-227ea, HFC-23, HFC-236fa, HFC-245fa, HFC-32, HFC-365mfc, HFC-41, HFC-43-10-mee, NF_3_ and SF_6_). A slightly different approach is used for emissions from combustion and non-combustion processes as shown in Figure 10.

Figure 10: General approach used to produce the GHG emission extensions

Acronyms: EP: energy product; EF: energy flow; EREU; emission-relevant energy use; I: industries, S: sources

The calculation of the combustion emissions follows the approach used for energy. The starting point for this type of emissions is the emission-relevant energy use data, which is multiplied by pollutant- and sector -specific emission factors from the IPCC (IPCC, 2006). The resulting air emission data is allocated to FIGARO-E3 industries and final consumption categories exactly the same way as in the case of energy. Emissions from non-combustion processes have been obtained directly from the EDGAR database (Crippa et al., 2021). In its public release, EDGAR provides information on emissions from 37 sources, ten of which refer to combustion processes (computed differently) and two to other categories not considered. Among those considered, non-combustion processes include the following categories: fugitive emissions from fuels, industrial processes and product use, agriculture and waste, which can be subdivided in 25 sources. Here, we used a more disaggregated version of the EDGAR database (made exclusively available for the project in which FIGARO-E3 has been built) that represents 117 different emission sources of non-combustion processes. The emission data has been allocated to industries using their output as proxy for relevant activity. The data provided aggregated all F-gases in one category based on GWP100 factors from (IPCC, 2014). The allocation tables from EDGAR sources to FIGARO-E3 industries is shown in the supplementary material.

In a last step, the data for European countries is benchmarked to the official emission accounts published by Eurostat (Eurostat, 2022a). Eurostat accounts provide the emission data of the same GHG categories used here (CO_2_, CH_4_, N_2_O and F-gases) for 64 industries. In order to maintain consistency with this source, the sum of the combustion and non-combustion emissions of European countries has been reconciled with the Eurostat data using a correspondence table (see supplementary material). Consequently, aggregating the GHG emissions from the 176 FIGARO-E3 industries to the 64 Eurostat industries yields the original Eurostat data. Below that level of aggregation, the subindustry proportions (e.g. the multiple agricultural subindustries represented in FIGARO-E3) are the same as those obtained by adding the combustion and non-combustion emissions described in the previous paragraphs. In the case of non-European countries, the combustion and non-combustion emissions are not benchmarked to any dataset. Thus, they are based on IEA and EDGAR data.

### Classifications

Product resolution

| **code** | **description** | **Analogous EXIO code** |
| --- | --- | --- |
| CPA_A01_A | Paddy rice | C_PARI |
| CPA_A01_B | Wheat | C_WHEA |
| CPA_A01_C | Cereal grains nec | C_OCER |
| CPA_A01_D | Vegetables, fruit, nuts | C_FVEG |
| CPA_A01_E | Oil seeds | C_OILS |
| CPA_A01_F | Sugar cane, sugar beet | C_SUGB |
| CPA_A01_G | Plant-based fibers | C_FIBR |
| CPA_A01_H | Crops nec | C_OTCR |
| CPA_A01_I | Cattle | C_CATL |
| CPA_A01_J | Pigs | C_PIGS |
| CPA_A01_K | Poultry | C_PLTR |
| CPA_A01_L | Meat animals nec | C_OMEA |
| CPA_A01_M | Animal products nec | C_OANP |
| CPA_A01_N | Raw milk | C_MILK |
| CPA_A01_O | Wool, silk-worm cocoons | C_WOOL |
| CPA_A01_P | Manure (conventional treatment) | C_MANC |
| CPA_A01_Q | Manure (biogas treatment) | C_MANB |
| CPA_A02 | Products of forestry, logging and related services | C_FORE |
| CPA_A03 | Fish and other fishing products, services incidental of fishing | C_FISH |
| CPA_B05_A | Anthracite | C_ANTH |
| CPA_B05_B | Coking Coal | C_COKC |
| CPA_B05_C | Other Bituminous Coal | C_OTBC |
| CPA_B05_D | Sub-Bituminous Coal | C_SUBC |
| CPA_B05_E | Patent Fuel | C_PATF |
| CPA_B05_F | Lignite/Brown Coal | C_LIBC |
| CPA_B05_G | BKB/Peat Briquettes | C_BKBP |
| CPA_B05_H | Peat | C_PEAT |
| CPA_B06_A | Crude petroleum and services related to crude oil extraction, excluding surveying | C_COIL |
| CPA_B06_B | Natural gas and services related to natural gas extraction, excluding surveying | C_GASE |
| CPA_B06_C | Natural Gas Liquids | C_GASL |
| CPA_B06_D | Other Hydrocarbons | C_OGPL |
| CPA_B07_A | Iron ores | C_IRON |
| CPA_B07_B | Uranium and thorium ores | C_ORAN |
| CPA_B07_C | Copper ores and concentrates | C_COPO |
| CPA_B07_D | Nickel ores and concentrates | C_NIKO |
| CPA_B07_E | Aluminium ores and concentrates | C_ALUO |
| CPA_B07_F | Precious metal ores and concentrates | C_PREO |
| CPA_B07_G | Lead, zinc and tin ores and concentrates | C_LZTO |
| CPA_B07_H | Other non-ferrous metal ores and concentrates | C_ONFO |
| CPA_B08_A | Stone | C_STON |
| CPA_B08_B | Sand and clay | C_SDCL |
| CPA_B08_C | Chemical and fertilizer minerals, salt and other mining and quarrying products n.e.c. | C_CHMF |
| CPA_C10_A | Products of meat cattle | C_PCAT |
| CPA_C10_B | Products of meat pigs | C_PPIG |
| CPA_C10_C | Products of meat poultry | C_PPLT |
| CPA_C10_D | Meat products nec | C_POME |
| CPA_C10_E | Fish products | C_FSHP |
| CPA_C10_F | Products of vegetable oils and fats | C_VOIL |
| CPA_C10_G | Dairy products | C_DAIR |
| CPA_C10_H | Processed rice | C_RICE |
| CPA_C10_I | Sugar | C_SUGR |
| CPA_C10_J | Food products nec | C_OFOD |
| CPA_C11 | Beverages | C_BEVR |
| CPA_C12 | Tobacco products | C_TOBC |
| CPA_C13 | Textiles | C_TEXT |
| CPA_C14 | Wearing apparel, furs | C_GARM |
| CPA_C15 | Leather and leather products | C_LETH |
| CPA_C16_A | Wood and products of wood and cork (except furniture), articles of straw and plaiting materials | C_WOOD |
| CPA_C16_B | Wood material for treatment, Re-processing of secondary wood material into new wood material | C_WOOW |
| CPA_C17_A | Pulp | C_PULP |
| CPA_C17_B | Paper and paper products | C_PAPE |
| CPA_C17_C | Secondary paper for treatment, Re-processing of secondary paper into new pulp | C_PAPR |
| CPA_C18 | Printing and recording services | C_PRIN |
| CPA_C191_A | Coke Oven Coke | C_COKE |
| CPA_C191_B | Gas Coke | C_GCOK |
| CPA_C191_C | Coal Tar | C_COTA |
| CPA_C192_A | Motor Gasoline | C_MGSL |
| CPA_C192_B | Aviation Gasoline | C_AGSL |
| CPA_C192_C | Gasoline Type Jet Fuel | C_GJET |
| CPA_C192_D | Kerosene Type Jet Fuel | C_KJET |
| CPA_C192_E | Kerosene | C_KERO |
| CPA_C192_F | Gas/Diesel Oil | C_DOIL |
| CPA_C192_G | Heavy Fuel Oil | C_FOIL |
| CPA_C192_H | Refinery Gas | C_RGAS |
| CPA_C192_I | Liquefied Petroleum Gases (LPG) | C_LPGA |
| CPA_C192_J | Refinery Feedstocks | C_REFF |
| CPA_C192_K | Ethane | C_ETHA |
| CPA_C192_L | Naphtha | C_NAPT |
| CPA_C192_M | White Spirit & SBP | C_WHSP |
| CPA_C192_N | Lubricants | C_LUBR |
| CPA_C192_O | Bitumen | C_BITU |
| CPA_C192_P | Paraffin Waxes | C_PARW |
| CPA_C192_Q | Petroleum Coke | C_PETC |
| CPA_C192_R | Non-specified Petroleum Products | C_NSPP |
| CPA_C20_A | N-fertiliser | C_NFER |
| CPA_C20_B | P- and other fertiliser | C_PFER |
| CPA_C20_C | Plastics, basic | C_PLAS |
| CPA_C20_D | Secondary plastic for treatment, Re-processing of secondary plastic into new plastic | C_PLAW |
| CPA_C20_E | Charcoal | C_CHAR |
| CPA_C20_F | Additives/Blending Components | C_ADDC |
| CPA_C20_G | Biogasoline | C_BIOG |
| CPA_C20_H | Biodiesels | C_BIOD |
| CPA_C20_I | Other Liquid Biofuels | C_OBIO |
| CPA_C21 | Chemicals nec | C_CHEM |
| CPA_C22 | Rubber and plastic products | C_RUBP |
| CPA_C23_A | Glass and glass products | C_GLAS |
| CPA_C23_B | Secondary glass for treatment, Re-processing of secondary glass into new glass | C_GLAW |
| CPA_C23_C | Bricks, tiles and construction products, in baked clay | C_BRIK |
| CPA_C23_D | Ceramic goods | C_CRMC |
| CPA_C23_E | Cement, lime and plaster | C_CMNT |
| CPA_C23_F | Ash for treatment, Re-processing of ash into clinker | C_ASHW |
| CPA_C23_G | Other non-metallic mineral products | C_ONMM |
| CPA_C24_A | Basic iron and steel and of ferro-alloys and first products thereof | C_STEL |
| CPA_C24_B | Secondary steel for treatment, Re-processing of secondary steel into new steel | C_STEW |
| CPA_C24_C | Precious metals | C_PREM |
| CPA_C24_D | Secondary preciuos metals for treatment, Re-processing of secondary preciuos metals into new preciuos metals | C_PREW |
| CPA_C24_E | Aluminium and aluminium products | C_ALUM |
| CPA_C24_F | Secondary aluminium for treatment, Re-processing of secondary aluminium into new aluminium | C_ALUW |
| CPA_C24_G | Lead, zinc and tin and products thereof | C_LZTP |
| CPA_C24_H | Secondary lead for treatment, Re-processing of secondary lead into new lead | C_LZTW |
| CPA_C24_I | Copper products | C_COPP |
| CPA_C24_J | Secondary copper for treatment, Re-processing of secondary copper into new copper | C_COPW |
| CPA_C24_K | Other non-ferrous metal products | C_ONFM |
| CPA_C24_L | Secondary other non-ferrous metals for treatment, Re-processing of secondary other non-ferrous metals into new other non-ferrous metals | C_ONFW |
| CPA_C24_M | Nuclear fuel | C_NUCF |
| CPA_C24_N | Foundry work services | C_METC |
| CPA_C25 | Fabricated metal products, except machinery and equipment | C_FABM |
| CPA_C26_A | Radio, television and communication equipment and apparatus | C_RATV |
| CPA_C26_B | Medical, precision and optical instruments, watches and clocks | C_MEIN |
| CPA_C27 | Electrical machinery and apparatus n.e.c. | C_ELMA |
| CPA_C28_A | Machinery and equipment n.e.c. | C_MACH |
| CPA_C28_B | Office machinery and computers | C_OFMA |
| CPA_C29 | Motor vehicles, trailers and semi-trailers | C_MOTO |
| CPA_C30 | Other transport equipment | C_OTRE |
| CPA_C31_32 | Furniture and other manufactured goods | C_FURM |
| CPA_C33 | Repair and installation services of machinery and equipment | C_REPA |
| CPA_D3511_A | Electricity by coal | C_POWC |
| CPA_D3511_B | Electricity by gas | C_POWG |
| CPA_D3511_C | Electricity by nuclear | C_POWN |
| CPA_D3511_D | Electricity by hydro | C_POWH |
| CPA_D3511_E | Electricity by wind | C_POWW |
| CPA_D3511_F | Electricity by petroleum and other oil derivatives | C_POWP |
| CPA_D3511_G | Electricity by biomass and waste | C_POWB |
| CPA_D3511_H | Electricity by solar photovoltaic | C_POWS |
| CPA_D3511_I | Electricity by solar thermal | C_POWE |
| CPA_D3511_J | Electricity by tide, wave, ocean | C_POWO |
| CPA_D3511_K | Electricity by Geothermal | C_POWM |
| CPA_D3511_L | Electricity nec | C_POWZ |
| CPA_D3512 | Transmission services of electricity | C_POWT |
| CPA_D3513 | Distribution and trade services of electricity | C_POWD |
| CPA_D352_A | Coke oven gas | C_COOG |
| CPA_D352_B | Blast Furnace Gas | C_MBFG |
| CPA_D352_C | Oxygen Steel Furnace Gas | C_MOSG |
| CPA_D352_D | Gas Works Gas | C_MGWG |
| CPA_D352_E | Biogas | C_MBIO |
| CPA_D352_F | Distribution services of gaseous fuels through mains | C_GASD |
| CPA_D353 | Steam and hot water supply services | C_HWAT |
| CPA_E36 | Collected and purified water, distribution services of water | C_WATR |
| CPA_E37T39_A | Secondary raw materials | C_RYMS |
| CPA_E37T39_B | Bottles for treatment, Recycling of bottles by direct reuse | C_BOTW |
| CPA_E37T39_C | Food waste for treatment; incineration | C_INCF |
| CPA_E37T39_D | Paper waste for treatment; incineration | C_INCP |
| CPA_E37T39_E | Plastic waste for treatment; incineration | C_INCL |
| CPA_E37T39_F | Intert/metal waste for treatment; incineration | C_INCM |
| CPA_E37T39_G | Textiles waste for treatment; incineration | C_INCT |
| CPA_E37T39_H | Wood waste for treatment; incineration | C_INCW |
| CPA_E37T39_I | Oil/hazardous waste for treatment; incineration | C_INCO |
| CPA_E37T39_J | Food waste for treatment; biogasification and land application | C_BIOF |
| CPA_E37T39_K | Paper waste for treatment; biogasification and land application | C_BIOP |
| CPA_E37T39_L | Sewage sludge for treatment; biogasification and land application | C_BIOS |
| CPA_E37T39_M | Food waste for treatment; composting and land application | C_COMF |
| CPA_E37T39_N | Paper and wood waste for treatment; composting and land application | C_COMW |
| CPA_E37T39_O | Food waste for treatment; waste water treatment | C_WASF |
| CPA_E37T39_P | Other waste for treatment; waste water treatment | C_WASO |
| CPA_E37T39_Q | Food waste for treatment; landfill | C_LANF |
| CPA_E37T39_R | Paper for treatment; landfill | C_LANP |
| CPA_E37T39_S | Plastic waste for treatment; landfill | C_LANL |
| CPA_E37T39_T | Inert/metal/hazardous waste for treatment; landfill | C_LANI |
| CPA_E37T39_U | Textiles waste for treatment; landfill | C_LANT |
| CPA_E37T39_V | Wood waste for treatment; landfill | C_LANW |
| CPA_F_A | Construction work | C_CONS |
| CPA_F_B | Secondary construction material for treatment, Re-processing of secondary construction material into aggregates | C_CONW |
| CPA_G45 | Sale, maintenance, repair of motor vehicles, motor vehicles parts, motorcycles, motor cycles parts and accessoiries | C_TDMO |
| CPA_G46 | Wholesale trade and commission trade services, except of motor vehicles and motorcycles | C_TDWH |
| CPA_G47_A | Retail trade services, except of motor vehicles and motorcycles | C_TDRT |
| CPA_G47_B | Retail trade services of motor fuel | C_TDFU |
| CPA_H49_A | Railway transportation services | C_TRAI |
| CPA_H49_B | Other land transportation services | C_TLND |
| CPA_H49_C | Transportation services via pipelines | C_TPIP |
| CPA_H50_A | Sea and coastal water transportation services | C_TWAS |
| CPA_H50_B | Inland water transportation services | C_TWAI |
| CPA_H51 | Air transport services | C_TAIR |
| CPA_H52 | Warehousing and support services for transportation | C_WARE |
| CPA_H53 | Postal and courier services | C_PTCR |
| CPA_I | Hotel and restaurant services | C_HORE |
| CPA_J58 | Publishing services | C_PUBS |
| CPA_J59_60 | Motion picture, video and television programme production services, sound recording and music publishing, programming and broadcasting | C_MOTI |
| CPA_J61 | Telecommunications services | C_TELC |
| CPA_J62_63 | Computer and related services | C_COMP |
| CPA_K64 | Financial intermediation services, except insurance and pension funding services | C_FINT |
| CPA_K65 | Insurance and pension funding services, except compulsory social security services | C_FINS |
| CPA_K66 | Services auxiliary to financial intermediation | C_FAUX |
| CPA_L | Real estate services | C_REAL |
| CPA_M69_70 | Legal and accounting services, services of head offices, management consultancy services | C_LGAC |
| CPA_M71 | Architectural and engineering services, technical testing and analysis services | C_ARCH |
| CPA_M72 | Research and development services | C_RESD |
| CPA_M73 | Advertising and market research services | C_ADMR |
| CPA_M74_75 | Other professional, scientific and technical services and veterinary services | C_OPRO |
| CPA_N77 | Renting services of machinery and equipment without operator and of personal and household goods | C_MARE |
| CPA_N78 | Employment services | C_EMPL |
| CPA_N79 | Travel agency, tour operator and other reservation services and related services | C_TRAV |
| CPA_N80T82 | Security and investigation services, services to buildings and landscape, office administrative, office support and other business support services | C_SECU |
| CPA_O84 | Public administration and defence services, compulsory social security services | C_PADF |
| CPA_P85 | Education services | C_EDUC |
| CPA_Q86 | Human health services | C_HUMH |
| CPA_Q87_88 | Residential care services, social work services without accommodation | C_CARE |
| CPA_R90T92 | Creative, arts, entertainment, library, archive, museum, other cultural services, gambling and betting services | C_CREA |
| CPA_R93 | Sporting services and amusement and recreation services | C_SPOR |
| CPA_S94 | Membership organisation services n.e.c. | C_ORGA |
| CPA_S95 | Repair services of personal and household goods | C_RCMP |
| CPA_S96 | Other services | C_OSER |
| CPA_T | Private households with employed persons | C_PRHH |
| CPA_U | Extra-territorial organizations and bodies | C_EXTO |

Industry resolution

| **code** | **description** | **Analogous EXIO code** |
| --- | --- | --- |
| A01_A | Cultivation of paddy rice | A_PARI |
| A01_B | Cultivation of wheat | A_WHEA |
| A01_C | Cultivation of cereal grains nec | A_OCER |
| A01_D | Cultivation of vegetables, fruit, nuts | A_FVEG |
| A01_E | Cultivation of oil seeds | A_OILS |
| A01_F | Cultivation of sugar cane, sugar beet | A_SUGB |
| A01_G | Cultivation of plant-based fibers | A_FIBR |
| A01_H | Cultivation of crops nec | A_OTCR |
| A01_I | Cattle farming | A_CATL |
| A01_J | Pigs farming | A_PIGS |
| A01_K | Poultry farming | A_PLTR |
| A01_L | Meat animals nec | A_OMEA |
| A01_M | Animal products nec | A_OANP |
| A01_N | Raw milk | A_MILK |
| A01_O | Wool, silk-worm cocoons | A_WOOL |
| A01_P | Manure treatment (conventional), storage and land application | A_MANC |
| A01_Q | Manure treatment (biogas), storage and land application | A_MANB |
| A02 | Forestry, logging and related service activities | A_FORE |
| A03 | Fishing, operating of fish hatcheries and fish farms, service activities incidental to fishing | A_FISH |
| B05 | Mining of coal and lignite, extraction of peat | A_COAL |
| B06_A | Extraction of crude petroleum and services related to crude oil extraction, excluding surveying | A_COIL |
| B06_B | Extraction of natural gas and services related to natural gas extraction, excluding surveying | A_GASE |
| B06_C | Extraction, liquefaction, and regasification of other petroleum and gaseous materials | A_OGPL |
| B07_A | Mining of iron ores | A_IRON |
| B07_B | Mining of uranium and thorium ores | A_ORAN |
| B07_C | Mining of copper ores and concentrates | A_COPO |
| B07_D | Mining of nickel ores and concentrates | A_NIKO |
| B07_E | Mining of aluminium ores and concentrates | A_ALUO |
| B07_F | Mining of precious metal ores and concentrates | A_PREO |
| B07_G | Mining of lead, zinc and tin ores and concentrates | A_LZTO |
| B07_H | Mining of other non-ferrous metal ores and concentrates | A_ONFO |
| B08_A | Quarrying of stone | A_STON |
| B08_B | Quarrying of sand and clay | A_SDCL |
| B08_C | Mining of chemical and fertilizer minerals, production of salt, other mining and quarrying n.e.c. | A_CHMF |
| C10_A | Processing of meat cattle | A_PCAT |
| C10_B | Processing of meat pigs | A_PPIG |
| C10_C | Processing of meat poultry | A_PPLT |
| C10_D | Production of meat products nec | A_POME |
| C10_E | Manufacture of fish products | A_FSHP |
| C10_F | Processing vegetable oils and fats | A_VOIL |
| C10_G | Processing of dairy products | A_DAIR |
| C10_H | Processed rice | A_RICE |
| C10_I | Sugar refining | A_SUGR |
| C10_J | Processing of Food products nec | A_OFOD |
| C11 | Manufacture of beverages | A_BEVR |
| C12 | Manufacture of tobacco products | A_TOBC |
| C13 | Manufacture of textiles | A_TEXT |
| C14 | Manufacture of wearing apparel, dressing and dyeing of fur | A_GARM |
| C15 | Tanning and dressing of leather, manufacture of luggage, handbags, saddlery, harness and footwear | A_LETH |
| C16_A | Manufacture of wood and of products of wood and cork, except furniture, manufacture of articles of straw and plaiting materials | A_WOOD |
| C16_B | Re-processing of secondary wood material into new wood material | A_WOOW |
| C17_A | Pulp | A_PULP |
| C17_B | Paper | A_PAPE |
| C17_C | Re-processing of secondary paper into new pulp | A_PAPR |
| C18 | Printing and reproduction of recorded media | A_PRIN |
| C191 | Manufacture of coke oven products | A_COKE |
| C192 | Petroleum Refinery | A_REFN |
| C20_A | N-fertiliser | A_NFER |
| C20_B | P- and other fertiliser | A_PFER |
| C20_C | Plastics, basic | A_PLAS |
| C20_D | Re-processing of secondary plastic into new plastic | A_PLAW |
| C21 | Chemicals nec | A_CHEM |
| C22 | Manufacture of rubber and plastic products | A_RUBP |
| C23_A | Manufacture of glass and glass products | A_GLAS |
| C23_B | Re-processing of secondary glass into new glass | A_GLAW |
| C23_C | Manufacture of bricks, tiles and construction products, in baked clay | A_BRIK |
| C23_D | Manufacture of ceramic goods | A_CRMC |
| C23_E | Manufacture of cement, lime and plaster | A_CMNT |
| C23_F | Re-processing of ash into clinker | A_ASHW |
| C23_G | Manufacture of other non-metallic mineral products n.e.c. | A_ONMM |
| C24_A | Manufacture of basic iron and steel and of ferro-alloys and first products thereof | A_STEL |
| C24_B | Re-processing of secondary steel into new steel | A_STEW |
| C24_C | Precious metals production | A_PREM |
| C24_D | Re-processing of secondary preciuos metals into new preciuos metals | A_PREW |
| C24_E | Aluminium production | A_ALUM |
| C24_F | Re-processing of secondary aluminium into new aluminium | A_ALUW |
| C24_G | Lead, zinc and tin production | A_LZTP |
| C24_H | Re-processing of secondary lead into new lead, zinc and tin | A_LZTW |
| C24_I | Copper production | A_COPP |
| C24_J | Re-processing of secondary copper into new copper | A_COPW |
| C24_K | Other non-ferrous metal production | A_ONFM |
| C24_L | Re-processing of secondary other non-ferrous metals into new other non-ferrous metals | A_ONFW |
| C24_M | Processing of nuclear fuel | A_NUCF |
| C24_N | Casting of metals | A_METC |
| C25 | Manufacture of fabricated metal products, except machinery and equipment | A_FABM |
| C26_A | Manufacture of radio, television and communication equipment and apparatus | A_RATV |
| C26_B | Manufacture of medical, precision and optical instruments, watches and clocks | A_MEIN |
| C27 | Manufacture of electrical machinery and apparatus n.e.c. | A_ELMA |
| C28_A | Manufacture of machinery and equipment n.e.c. | A_MACH |
| C28_B | Manufacture of office machinery and computers | A_OFMA |
| C29 | Manufacture of motor vehicles, trailers and semi-trailers | A_MOTO |
| C30 | Manufacture of other transport equipment | A_OTRE |
| C31_32 | Manufacture of furniture, other manufacturing | A_FURM |
| C33 | Repair and installation of machinery and equipment | A_REPA |
| D3511_A | Production of electricity by coal | A_POWC |
| D3511_B | Production of electricity by gas | A_POWG |
| D3511_C | Production of electricity by nuclear | A_POWN |
| D3511_D | Production of electricity by hydro | A_POWH |
| D3511_E | Production of electricity by wind | A_POWW |
| D3511_F | Production of electricity by petroleum and other oil derivatives | A_POWP |
| D3511_G | Production of electricity by biomass and waste | A_POWB |
| D3511_H | Production of electricity by solar photovoltaic | A_POWS |
| D3511_I | Production of electricity by solar thermal | A_POWE |
| D3511_J | Production of electricity by tide, wave, ocean | A_POWO |
| D3511_K | Production of electricity by Geothermal | A_POWM |
| D3511_L | Production of electricity nec | A_POWZ |
| D3512 | Transmission of electricity | A_POWT |
| D3513 | Distribution and trade of electricity | A_POWD |
| D352 | Manufacture of gas, distribution of gaseous fuels through mains | A_GASD |
| D353 | Steam and hot water supply | A_HWAT |
| E36 | Collection, purification and distribution of water | A_WATR |
| E37T39_A | Recycling of waste and scrap | A_RYMS |
| E37T39_B | Recycling of bottles by direct reuse | A_BOTW |
| E37T39_C | Incineration of waste; Food | A_INCF |
| E37T39_D | Incineration of waste; Paper | A_INCP |
| E37T39_E | Incineration of waste; Plastic | A_INCL |
| E37T39_F | Incineration of waste; Metals and Inert materials | A_INCM |
| E37T39_G | Incineration of waste; Textiles | A_INCT |
| E37T39_H | Incineration of waste; Wood | A_INCW |
| E37T39_I | Incineration of waste; Oil/Hazardous waste | A_INCO |
| E37T39_J | Biogasification of food waste, incl. land application | A_BIOF |
| E37T39_K | Biogasification of paper, incl. land application | A_BIOP |
| E37T39_L | Biogasification of sewage slugde, incl. land application | A_BIOS |
| E37T39_M | Composting of food waste, incl. land application | A_COMF |
| E37T39_N | Composting of paper and wood, incl. land application | A_COMW |
| E37T39_O | Waste water treatment, food | A_WASF |
| E37T39_P | Waste water treatment, other | A_WASO |
| E37T39_Q | Landfill of waste; Food | A_LANF |
| E37T39_R | Landfill of waste; Paper | A_LANP |
| E37T39_S | Landfill of waste; Plastic | A_LANL |
| E37T39_T | Landfill of waste; Inert/metal/hazardous | A_LANI |
| E37T39_U | Landfill of waste; Textiles | A_LANT |
| E37T39_V | Landfill of waste; Wood | A_LANW |
| F_A | Construction | A_CONS |
| F_B | Re-processing of secondary construction material into aggregates | A_CONW |
| G45 | Sale, maintenance, repair of motor vehicles, motor vehicles parts, motorcycles, motor cycles parts and accessoiries | A_TDMO |
| G46 | Wholesale trade and commission trade, except of motor vehicles and motorcycles | A_TDWH |
| G47_A | Retail trade, except of motor vehicles and motorcycles | A_TDRT |
| G47_B | Retail sale of automotive fuel | A_TDFU |
| H49_A | Transport via railways | A_TRAI |
| H49_B | Other land transport | A_TLND |
| H49_C | Transport via pipelines | A_TPIP |
| H50_A | Sea and coastal water transport | A_TWAS |
| H50_B | Inland water transport | A_TWAI |
| H51 | Air transport | A_TAIR |
| H52 | Warehousing and support activities for transportation | A_WARE |
| H53 | Postal and courier activities | A_PTCR |
| I | Hotels and restaurants | A_HORE |
| J58 | Publishing activities | A_PUBS |
| J59_60 | Motion picture, video, television programme production, programming and broadcasting activities | A_MOTI |
| J61 | Telecommunications | A_TELC |
| J62_63 | Computer and related services | A_COMP |
| K64 | Financial intermediation, except insurance and pension funding | A_FINT |
| K65 | Insurance and pension funding, except compulsory social security | A_FINS |
| K66 | Activities auxiliary to financial intermediation | A_FAUX |
| L | Real estate activities | A_REAL |
| M69_70 | Legal and accounting activities, activities of head offices, management consultancy activities | A_LGAC |
| M71 | Architectural and engineering activities, technical testing and analysis | A_ARCH |
| M72 | Research and development | A_RESD |
| M73 | Advertising and market research | A_ADMR |
| M74_75 | Other professional, scientific and technical activities, veterinary activities | A_OPRO |
| N77 | Renting of machinery and equipment without operator and of personal and household goods | A_MARE |
| N78 | Employment activities | A_EMPL |
| N79 | Travel agency, tour operator reservation service and related activities | A_TRAV |
| N80T82 | Security and investigation, service and landscape, office administrative and support activities | A_SECU |
| O84 | Public administration and defence, compulsory social security | A_PADF |
| P85 | Education | A_EDUC |
| Q86 | Human health activities | A_HUMH |
| Q87_88 | Residential care activities and social work activities without accommodation | A_CARE |
| R90T92 | Creative, arts and entertainment activities, libraries, archives, museums and other cultural activities, gambling and betting activities | A_CREA |
| R93 | Sports activities and amusement and recreation activities | A_SPOR |
| S94 | Activities of membership organisation n.e.c. | A_ORGA |
| S95 | Repair services of personal and household goods | A_RCMP |
| S96 | Other service activities | A_OSER |
| T | Private households with employed persons | A_PRHH |
| U | Extra-territorial organizations and bodies | A_EXTO |

**References**

Ark, B. van, & Jäger, K. (2017). Recent Trends in Europe’s Output and Productivity Growth Performance at the Sector Level, 2002-2015. *International Productivity Monitor*, *33*(Fall 2017).

Banfi, S., Filippini, M., & Hunt, L. C. (2005). Fuel tourism in border regions: The case of Switzerland. *Energy Economics*, *27*(5), 689–707. https://doi.org/10.1016/j.eneco.2005.04.006

Clarke, D., Flachenecker, F., Guidetti, E., & Pionnier, P.-A. (2022). *CO2 Emissions from air transport—A near-real-time global database for policy analysis*.

Crippa, M., Guizzardi, D., Solazzo, E., Muntean, M., Schaaf, E., M. Monforti-Ferrario, F. B., Olivier, J. G. J., Grassi, G., Rossi, S., & Vignati, E. (2021). *GHG emissions of all world countries—2021 Report*. Joint Research Centre.

de la Torre Cuevas, F., Pereira, X., & López-Iglesias, E. (2023). A new alternative for matrix balancing under conflicting information. *Economic Systems Research*, 1–27. https://doi.org/10.1080/09535314.2023.2170217

EC. (2016). *Eurostat*. http://ec.europa.eu/eurostat

Eurostat. (2008). *Eurostat Manual of Supply, Use and Input-Output Tables*. Eurostat.

Eurostat. (2022a). *Air emissions accounts by NACE Rev. 2 activity (env_ac_ainah_r2)*. Eurostat.

Eurostat. (2022b). *Air emissions accounts totals bridging to emission inventory totals (env_ac_aibrid_r2)*.

IEA. (2017a). *Energy Balances of non-OECD Countries (2016 edition)*. International Energy Agency.

IEA. (2017b). *Energy Balances of OECD Countries (2016 edition)*. International Energy Agency.

IEA. (2017c). *World Energy Balances. Documentation for Beyond 2020 Files (2016 Edition)*. International Energy Agency.

ILO. (2012). *ISCO-08—International Standard Classification of Occupations*. www.ilo.org/public/english/bureau/stat/isco

ILO. (2013). *Guide to the new Millennium Development Goals Employment indicators: Including the full decent work indicator set*. www.ilo.org/wcmsp5/groups/public/—ed_emp/documents/publication/wcms_11051

ILO. (2014). *ILOSTAT Database.* www.ilo.org/ilostat/faces/oracle/webcenter/portalapp/pagehierarchy/Page137.jspx?_afrLoop=96793898991792&clean=true#!%40%40%3F_afrLoop%3D96793898991792%26clean%3Dtrue%26_adf.ctrl-state%3Dmn1×63v2o_9

IPCC. (2006). *2006 IPCC Guidelines for National Greenhouse Gas Inventories. Volume 2: Energy*. Institute for Global Environmental Strategies.

IPCC. (2014). *Climate Change 2014: Synthesis Report. Contribution of Working Groups I, II and III to the Fifth Assessment Report of the Intergovernmental Panel on Climate Change*. Intergovernmental Panel on Climate Change.

Jäger, K. (2017). *Eu klems growth and productivity accounts 2017 release (Revised July 2018), statistical module1*.

Jansen, D.-J., & Jonker, N. (2018). Fuel tourism in Dutch border regions: Are only salient price differentials relevant? *Energy Economics*, *74*, 143–153. https://doi.org/10.1016/j.eneco.2018.05.036

Lenzen, M., Gallego, B., & Wood, R. (2009). Matrix balancing under conflicting information. *Economic Systems Research*, *21*(1), 23–44. https://doi.org/10.1080/09535310802688661

Lenzen, M., Geschke, A., West, J., Fry, J., Malik, A., Giljum, S., Milà i Canals, L., Piñero, P., Lutter, S., Wiedmann, T., Li, M., Sevenster, M., Potočnik, J., Teixeira, I., Van Voore, M., Nansai, K., & Schandl, H. (2022). Implementing the material footprint to measure progress towards Sustainable Development Goals 8 and 12. *Nature Sustainability*, *5*(2), 157–166. https://doi.org/10.1038/s41893-021-00811-6

OECD. (2014). *OECD statistics*. http://stats.oecd.org/

OECD. (2023). *OECD Inter-Country Input-Output Database*. Organisation for Economic Co-operation and Development. http://oe.cd/icio

Rémond-Tiedrez, I., & Rueda-Cantuche, J. M. (2019). *EU inter-country supply, use and input-output tables: Full international and global accounts for research in input-output analysis (FIGARO): 2019 edition*. European Commission.

Simas, M. S., Golsteijn, L., Huijbregts, M. A. J., Wood, R., & Hertwich, E. G. (2014). The ‘bad labor’ footprint: Quantifying the social impacts of globalization. *Sustainability (Switzerland)*, *6*(11), 7514–7540. https://doi.org/10.3390/su6117514

Simas, M., Wood, R., & Hertwich, E. (2014). Labor Embodied in Trade. *Journal of Industrial Ecology*, *19*(3), 343–356. https://doi.org/10.1111/jiec.12187

Stadler, K., Wood, R., Bulavskaya, T., Södersten, C.-J., Simas, M., Schmidt, S., Usubiaga, A., Acosta-Fernández, J., Kuenen, J., Bruckner, M., Giljum, S., Lutter, S., Merciai, S., Schmidt, J. H., Theurl, M. C., Plutzar, C., Kastner, T., Eisenmenger, N., Erb, K.-H., … Tukker, A. (2018a). Developing a time series of detailed Environmentally Extended Multi-Regional Input-Output tables. *Journal of Industrial Ecology*, *22*(3), 502–515.

Stadler, K., Wood, R., Bulavskaya, T., Södersten, C.-J., Simas, M., Schmidt, S., Usubiaga, A., Acosta-Fernández, J., Kuenen, J., Bruckner, M., Giljum, S., Lutter, S., Merciai, S., Schmidt, J. H., Theurl, M. C., Plutzar, C., Kastner, T., Eisenmenger, N., Erb, K.-H., … Tukker, A. (2018b). EXIOBASE 3: Developing a Time Series of Detailed Environmentally Extended Multi-Regional Input-Output Tables. *Journal of Industrial Ecology*, *22*(3), 502–515. https://doi.org/10.1111/jiec.12715

Usubiaga, A., & Acosta-Fernández, J. (2015). CARBON EMISSION ACCOUNTING IN MRIO MODELS: THE TERRITORY VS. THE RESIDENCE PRINCIPLE. *Economic Systems Research*, *27*(4), 458–477. https://doi.org/10.1080/09535314.2015.1049126

Usubiaga-Liaño, A., Arto, I., & Acosta-Fernández, J. (2021). Double accounting in energy footprint and related assessments: How common is it and what are the consequences? *Energy*, *222*, 119891. https://doi.org/10.1016/j.energy.2021.119891

Usubiaga-Liaño, A., Behrens, P., & Daioglou, V. (2020). Energy use in the global food system. *Journal of Industrial Ecology*, *n/a*(n/a). https://doi.org/10.1111/jiec.12982

Valderas-Jaramillo, J. M., & Rueda-Cantuche, J. M. (2021). The multidimensional nD-GRAS method: Applications for the projection of multiregional input–output frameworks and valuation matrices. *Papers in Regional Science*, *100*(6), 1599–1624. https://doi.org/10.1111/pirs.12625

Wlazlowski, S., Giulietti, M., Binner, J., & Milas, C. (2009). Price dynamics in European petroleum markets. *Energy Economics*, *31*(1), 99–108. https://doi.org/10.1016/j.eneco.2008.08.009

1. As also in STAN (STructural ANalysis Database – OECD), the variables EMPN and EMPE represent Headcounts or Number of jobs depending on availability while EMPN_FTE and EMPE_FTE contain Full-time equivalent jobs whenever available. HOURS contains any hours worked data available (ideally hours actually worked per person per year). [↑](#footnote-ref-1)
2. In principle, the OECD data is said to be provided for 45 industries, [see TiVA 2021 – Industries](https://www.oecd.org/industry/ind/TiVA-2021-industries.pdf), however in practice the detail for this variable is for 36 industries. Looking at the common classifications for countries of FIGARO-E3, we may observe the high consistency with the Eurostat data (“Employment by sex, age and economic activity”, provided from 2008 onwards, NACE Rev. 2). [↑](#footnote-ref-2)
3. According to this work, socioeconomic accounts in EXIOBASE 3 comprise total and vulnerable employment, both in persons and in hours. Vulnerable employment (ILO, 2013; M. Simas et al., 2014; M. S. Simas et al., 2014), consisting of unpaid family workers and self-employed persons, was also a new addition to the socioeconomic indicators in EXIOBASE 3. According to them, persons and hours worked accounts were based on available statistics from Eurostat (EC, 2016), the International Labour Organization’s (ILO) ILOSTAT database (ILO, 2014) and from the OECD’s Statistics (OECD, 2014), which were later on updated. [↑](#footnote-ref-3)
4. Total employment refers to total persons engaged in each industry in the MRIO. It covers both employees and self-employed persons. Definitions are given by the International Labour Organization (ILO). Employees are all persons with formal job attachment, even if in temporarily paid or unpaid leave; and self-employed persons include employers, own-account workers, members of producers’ cooperatives, unpaid family workers at work, and persons engaged in the production of economic goods and services for own and household consumption. Number of employees and self-employed are available in the socio-economic database, but not included in the model. [↑](#footnote-ref-4)
5. EXIOBASE accounts of A_FURN, A_TDTR, A_TAUX, A_PTEL, A_OBUS, A_HEAL, A_RECR. [↑](#footnote-ref-5)
6. http://www.euklems.net/ [↑](#footnote-ref-6)
7. https://ilostat.ilo.org/topics/employment/ [↑](#footnote-ref-7)
8. Still, data on skill levels (high-, medium- and low-skilled work) corresponded to that of the International Standard Classification of Occupations (ILO, 2012), collecting the socioeconomic indicators for the period from 1995 to 2011. It was only explicit that adjustments were made to disaggregate and combine different data sources and industry classifications throughout the period, as discussed on their annex report for labour accounts (supporting information S7 on the Web). [↑](#footnote-ref-8)
9. In practice, EXIOBASE structures need to be disaggregated to reach FIGARO-e classification (the same coefficients per unit of output are assumed for the subindustries), in particular: EXIO account A_MDIA: Publishing, printing and reproduction of recorded media (22), which is split in A_PRIN: Printing and reproduction of recorded media; A_PUBS: Publishing activities; EXIO account A_FURN: Manufacture of furniture; manufacturing n.e.c. (36), which is split in A_FURM: Manufacture of furniture; other manufacturing; A_REPA: Repair and installation of machinery and equipment; EXIO account A_TAUX: Supporting and auxiliary transport activities; activities of travel agencies (63), which is split in A_WARE: Warehousing and support activities for transportation; A_TRAV: Travel agency, tour operator reservation service and related activities; EXIO account A_PTEL: Post and telecommunications (64), which is split in A_PTCR: Postal and courier activities; A_TELC: Telecommunications; EXIO account A_OBUS: Other business activities (74), which is split in 6 subindustries; EXIO account A_HEAL: Health and social work (85), which is split in A_HUMH: Human health activities; A_CARE: Residential care activities and social work activities without accommodation; and EXIO account A_RECR: Recreational, cultural and sporting activities (92) which is split in three subindustries. [↑](#footnote-ref-9)
10. Based on the original gender and skill structure of EXIOBASE: “Employment_FIGAROe EXIOgendrSkl”

    Based on the gender and by skill totals from Occupations: “Employment_FIGAROe ICIO OOC”.

    Based on the gender and by skill totals from Education: “Employment_FIGAROe ICIO EDU”. [↑](#footnote-ref-10)
